# Replication catastrophe is responsible for intrinsic PAR glycohydrolase inhibitor-sensitivity in patient-derived ovarian cancer models

**DOI:** 10.1186/s13046-021-02124-0

**Published:** 2021-10-16

**Authors:** Camilla Coulson-Gilmer, Robert D. Morgan, Louisa Nelson, Bethany M. Barnes, Anthony Tighe, René Wardenaar, Diana C. J. Spierings, Helene Schlecht, George J. Burghel, Floris Foijer, Sudha Desai, Joanne C. McGrail, Stephen S. Taylor

**Affiliations:** 1 Division of Cancer Sciences, Faculty of Biology, Medicine and Health, University of Manchester, Manchester Cancer Research Centre, Oglesby Cancer Research Building, 555 Wilmslow Road, Manchester, M20 4GJ UK; 2 Department of Medical Oncology, The Christie NHS Foundation Trust, Wilmslow Rd, Manchester, M20 4BX UK; 3 European Research Institute for the Biology of Ageing (ERIBA), University of Groningen, University Medical Center Groningen, Groningen, 9713 AV The Netherlands; 4 Manchester Centre for Genomic Medicine, St Mary’s Hospital, Manchester University NHS Foundation Trust, Oxford Road, Manchester, M13 9WL UK; 5 Department of Histopathology, The Christie NHS Foundation Trust, Wilmslow Rd, Manchester, M20 4BX UK

**Keywords:** High-grade serous ovarian cancer, PARG inhibitor, DNA replication, Replication stress, Gene expression signature, Predictive biomarkers

## Abstract

**Background:**

Patients with ovarian cancer often present at advanced stage and, following initial treatment success, develop recurrent drug-resistant disease. PARP inhibitors (PARPi) are yielding unprecedented survival benefits for women with BRCA-deficient disease. However, options remain limited for disease that is platinum-resistant and/or has inherent or acquired PARPi-resistance. PARG, the PAR glycohydrolase that counterbalances PARP activity, is an emerging target with potential to selectively kill tumour cells harbouring oncogene-induced DNA replication and metabolic vulnerabilities. Clinical development of PARG inhibitors (PARGi) will however require predictive biomarkers, in turn requiring an understanding of their mode of action. Furthermore, differential sensitivity to PARPi is key for expanding treatment options available for patients.

**Methods:**

A panel of 10 ovarian cancer cell lines and a living biobank of patient-derived ovarian cancer models (OCMs) were screened for PARGi-sensitivity using short- and long-term growth assays. PARGi-sensitivity was characterized using established markers for DNA replication stress, namely replication fibre asymmetry, RPA foci, KAP1 and Chk1 phosphorylation, and pan-nuclear γH2AX, indicating DNA replication catastrophe. Finally, gene expression in sensitive and resistant cells was also examined using NanoString or RNAseq.

**Results:**

PARGi sensitivity was identified in both ovarian cancer cell lines and patient-derived OCMs, with sensitivity accompanied by markers of persistent replication stress, and a pre-mitotic cell cycle block. Moreover, DNA replication genes are down-regulated in PARGi-sensitive cell lines consistent with an inherent DNA replication vulnerability. However, DNA replication gene expression did not predict PARGi-sensitivity in OCMs. The subset of patient-derived OCMs that are sensitive to single-agent PARG inhibition, includes models that are PARPi- and/or platinum-resistant, indicating that PARG inhibitors may represent an alternative treatment strategy for women with otherwise limited therapeutic options.

**Conclusions:**

We discover that a subset of ovarian cancers are intrinsically sensitive to pharmacological PARG blockade, including drug-resistant disease, underpinned by a common mechanism of replication catastrophe. We explore the use of a transcript-based biomarker, and provide insight into the design of future clinical trials of PARGi in patients with ovarian cancer. However, our results highlight the complexity of developing a predictive biomarker for PARGi sensitivity.

**Supplementary Information:**

The online version contains supplementary material available at 10.1186/s13046-021-02124-0.

## Background

Ovarian cancer is a leading cause of gynaecological cancer-related death, accounting for more than 200,000 deaths globally in 2020 [1, 2]. The most prevalent subtype, high-grade serous ovarian cancer (HGSOC), accounts for approximately 70% of cases and is frequently lethal because it is highly aggressive and presents at an advanced stage where cure is unlikely [3]. Treatment options are limited, but typically include cytoreductive surgery plus paclitaxel/platinum chemotherapy, with maintenance therapy used in specific subgroups [4, 5]. While many patients initially respond well, most develop recurrent disease, yielding 10-year survival rates of only ~ 35% [6–9].

HGSOC is characterised by ubiquitous *TP53* mutations, and extensive copy number variation (CNV) [10, 11], implying chromosome instability [12], and indeed, HGSOC is one of the most chromosomally unstable cancers [6, 11, 13]. Approximately 50% have homologous recombination (HR) deficiency (HRD), which in ~ 20% of cases is due to alteration of *BRCA1* or *BRCA2* [14]. While targeted therapies have led to personalised treatments across many cancers, this therapeutic strategy is limited in HGSOC due to the paucity of actionable driver mutations. Other therapeutic strategies are therefore required and indeed, HRD opened up an alternative: synthetic lethality. This approach was pioneered by the ability of poly(ADP-ribose) polymerase (PARP) inhibitors (PARPi) to selectively kill *BRCA*-mutant cells [15, 16], and these drugs are now yielding major benefits for patients with HRD tumours [17–20]. However, this leaves ~ 50% of cases that are HR-proficient (HRP) and therefore unlikely to benefit from a PARPi. Furthermore, the majority of HRD tumours will eventually acquire PARPi resistance with treatment [21]. Thus, alternative strategies are required to improve outcomes for women with inherent and acquired resistance to both PARP inhibition and platinum-based chemotherapy.

To develop novel therapeutic strategies, attention is focussing on targeting poly(ADP-ribose) (PAR) glycohydrolase (PARG), the enzyme that counterbalances PARP1/2 activity [22–27]. Thus far, we have characterised PARG inhibitor PDD00017273, hereafter PARGi, a quinazolinedione that inhibits PARG with an in vitro IC_50_ of 26 nM [28]. Upon analysing a panel of six ovarian cancer cell lines in response to PARGi and the PARP inhibitor Olaparib, hereafter PARPi, we discovered that OVSAHO, COV318, COV362 and CAOV3 proliferated in both inhibitors, while Kuramochi and OVCAR3 displayed differential sensitivities; Kuramochi proliferation was suppressed by PARGi, while OVCAR3 proliferation was suppressed by PARPi [26]. Sensitivity of Kuramochi to PARGi was accompanied by pan-nuclear γH2AX staining, which is indicative of replication catastrophe [29], and a synthetic lethal siRNA screen identified several DNA replication genes, including *TIMELESS*, that when inhibited sensitised OVCAR3 to PARGi. Furthermore, PARGi induced replication fork asymmetry in Kuramochi but not OVCAR3, suggesting that Kuramochi cells have an underlying DNA replication vulnerability that causes frequent fork stalling, and are thus more reliant on PARG to re-start stalled forks, possibly by reversing PARP-mediated inhibition of RECQ1 [26].

The notion that DNA replication vulnerabilities might confer PARGi sensitivity led to the identification of additional PARGi-sensitive cell lines; interrogation of Cancer Cell Line Encyclopedia (CCLE) data identified several ovarian cancer cell lines with down-regulated DNA replication genes, namely HS571T, RMG1, OV56, OVMANA and OVISE, as well as Kuramochi [26]. While HS571T is no longer available for research purposes, we obtained RMG1, OV56, OVMANA and OVISE, and showed that RMG1 and OVMANA are PARGi-sensitive. This raised the possibility that a DNA “*replication stress*” gene expression signature might have potential as a predictive biomarker of PARGi sensitivity. Importantly, like Kuramochi, RMG1 showed differential sensitivity to PARGi and PARPi. However, while OVMANA were more sensitive to PARGi than PARPi, the distinction was less clear-cut.

These observations indicate that PARG inhibitors may open up new opportunities to treat ovarian cancers. However, a number of questions remained unanswered. Firstly, is the PARGi sensitivity exhibited by different cell lines mediated via the same mechanism? While PARGi prevents proliferation of RMG1 and OVMANA, whether this was due to the replication catastrophe phenomenon exhibited by Kuramochi cells was not established. Secondly, does PARGi sensitivity correlate with down-regulated DNA replication genes? While the CCLE data indicated that DNA replication genes are down-regulated in OV56 and OVISE, their PARGi sensitivity was unclear [26]. If resistant, the utility of a DNA “*replication stress*” gene expression signature as a predictive biomarker of PARGi sensitivity is uncertain. To address these two questions, we set out to perform a detailed analysis of a panel of ovarian cancer cell lines to determine (a) whether PARGi sensitivity is via a common replication catastrophe mechanism, and (b) whether PARGi sensitivity does indeed correlate with the relative expression levels of DNA replication genes. Moreover, to assess the translational opportunity of PARGi, we screened a panel of patient-derived ovarian cancer models (OCMs) [13] to determine whether any were PARGi sensitive and if this sensitivity is via a replication stress mechanism that also correlates with expression levels of DNA replication genes.

## Methods

### Materials

PDD00017273 (PARGi; Tocris Bioscience), PDD00031704 [PARGi-Me [28]], olaparib (PARPi; AZD2281, KU0059436; Selleckchem), niraparib tosylate (MK-4827, Zejula; Selleckchem) and paclitaxel (Sigma-Aldrich) were dissolved in DMSO. Cisplatin (Sigma-Aldrich) was dissolved in 0.9% NaCl. Hydroxyurea (Sigma-Aldrich) was dissolved in ddH_2_O. BrdU and IdU (Sigma-Aldrich) were dissolved in DMSO and culture media, respectively.

### Established cell lines

The ovarian cancer cell lines OVCAR3 (ATCC), Kuramochi, OVMANA, OVSAHO, OVISE (JCRB Cell Bank) were grown in RPMI; COV362, COV318 (Sigma-Aldrich) and CAOV3 (ATCC) were grown in DMEM; RMG1 (JCRB Cell Bank) was grown in Ham’s F12 (Sigma-Aldrich); all supplemented with 10% foetal bovine serum (FBS; Life Science Group), 100 U/ml streptomycin, 100 U/ml penicillin (Sigma-Aldrich), 2 mM glutamine (Sigma-Aldrich) and maintained at 37 °C in a humidified 5% CO_2_ atmosphere. OV56 (Sigma-Aldrich) was grown in DMEM/F12 with FBS reduced to 5% and supplemented with 10 μg/ml insulin, 0.5 μg/ml hydrocortisone, 100 U/ml streptomycin, 100 U/ml penicillin, 2 mM glutamine and maintained at 37 °C in a humidified 5% CO_2_ atmosphere. All ovarian cancer cell lines were authenticated by the Molecular Biology Core Facility at the CRUK Manchester Institute using Promega Powerplex 21 System, and underwent periodic testing for mycoplasma.

### Ex vivo ovarian cancer models (OCMs)

Research samples were obtained with informed patient consent from the Manchester Cancer Research Centre (MCRC) Biobank. The MCRC Biobank is licensed by the Human Tissue Authority (license number: 30004) and is ethically approved as a research tissue bank by the South Manchester Research Ethics Committee (Ref: 18/NW/0092). The role of the MCRC Biobank is to distribute samples and does not endorse studies performed or the interpretation of results. For more information, see https://www.mcrc.manchester.ac.uk/research/mcrc-biobank. Ex vivo ovarian cancer models (OCMs) were expanded from ascites samples from 32 patients, of which seven were published previously [13]. All patients were initially diagnosed with HGSOC, however four were re-classified to: cytological diagnosis of ‘suspicion of adenocarcinoma arising from the gynaecological tract’ (patients 87 and 195), moderately differentiated (intermediate grade/grade 2) serous adenocarcinoma (patient 152), and low-grade serous ovarian cancer (patient 64) [30]. The age at diagnosis ranged from 44 to 84 years, the mean age at diagnosis was 62.8 years. Five samples were chemonaïve (patients 87, 110, 99, 195 and 231).

Ovarian cancer and stromal cells from patients were cultured as previously described [13]. In brief, OCMI media [31] was used with a 50:50 mix of Nutrient Mixture Ham’s F12 and Medium 199 (Life Technologies) supplemented with 5% FBS or 5% Hyclone FBS (Cytiva), 2 mM glutamine, 100 U/ml penicillin, 100 U/ml streptomycin, 10 mM HEPES at pH 7.4, 20 μg/ml insulin, 0.01 μg/ml EGF, 0.5 μg/ml hydrocortisone, 10 μg/ml transferrin, 0.2 pg/ml Triiodothyronine, 5 μg/ml o-phosphorylethanolamine, 8 ng/ml selenious acid, 0.5 ng/ml 17β-oestradiol, 5 μg/ml all trans retinoic acid, 1.75 μg/ml hypoxanthine, 0.05 μg/ml lipoic acid, 0.05 μg/ml cholesterol, 0.012 μg/ml ascorbic acid, 0.003 μg/ml α-tocopherol phosphate, 0.025 μg/ml calciferol, 3.5 μg/ml choline chloride, 0.33 μg/ml folic acid, 0.35 μg/ml vitamin B_12_, 0.08 μg/ml thiamine HCL, 4.5 μg/ml i-inositol, 0.075 μg/ml uracil, 0.125 μg/ml ribose, 0.0125 μg/ml para-aminobenzoic acid, 1.25 mg/ml BSA, 0.085 μg/ml xanthine and 25 ng/ml cholera toxin (all from Sigma-Aldrich). To establish OCMs 500–1000 mL of ascitic fluid was centrifuged (500×g for 10 min at 4 °C) and cell pellets pooled in HBSS (Life Technologies). Red blood cells were removed using a red blood cell lysis buffer (Miltenyi Biotec) as per the manufacturer’s instructions. Tumour cells were seeded into Primaria flasks containing OCMI. Cultures were incubated for 2–4 days at 37 °C in a humidified 5% CO_2_ and 5% O_2_ atmosphere. Media was replaced every 3–4 days. Upon cell attachment, stromal cells were separated from the mixed sample using 0.05% trypsin-EDTA (Sigma-Aldrich) and seeded in gelatin-coated flasks in OCMI media containing 5% FBS (Life Science Group). Once tumour cells reached 95% confluency, cells were passaged using 0.25% Trypsin-EDTA, centrifuged in DMEM containing 20% FBS and re-plated at a 1:2 ratio.

### Colony formation assay

For established ovarian cancer cell lines, 1000 cells per well were seeded into 6-well plates 24 h prior to drug treatment. For OCMs, 2000 cells per well were seeded into either Primaria or collagen coated (50 μg/mL of BD collagen type I, rat tail, [BD Biosciences Discovery Labware] in 0.02 N Acetic Acid for 1 h prior to seeding) 6-well plates 24 h prior to drug treatment. For OCM.165, tumour cells were seeded at 4000 cells per well 24 h prior to drug treatment. DMSO (control), PARGi, PARGi-Me or PARPi were added; cells were treated either continuously or the drugs were washed out at the specific time points as indicated in the figures. Following colony formation (range: 2–4 weeks for ovarian cancer cell lines; range: 2–12 weeks for OCMs), colonies were fixed in 1% formaldehyde for 10 min, stained with 0.05% (w/v) crystal violet solution (Sigma-Aldrich) for 10 min and rinsed with ddH_2_O. Plates were imaged using a ChemiDoc™ Touch Imaging System (BioRad), and analysed with an ImageJ ‘colony area’ plug-in [32].

### Immunofluorescence

All cells were plated onto either 13 mm or 19 mm coverslips 24 h prior to drug treatment. For RMG1 cells, coverslips were coated with 0.01% Poly-L-Lysine (Sigma-Aldrich). For OCMs, coverslips were collagen coated (as above) prior to plating. OCMs were plated at 28,000 cells per coverslips (on 19 mm coverslips) for 24 h prior to drug treatment. OCM.165 was plated at 112,000 cells per coverslip. All OCMs were plated for 48 h prior to immunofluorescence staining for markers of HGSOC, including: p53, Paired-box gene 8 (PAX8) and cytokeratin 7 (CK7). For immunofluorescence staining, cells were washed with PBS (× 2), fixed in 1% formaldehyde for 5 min, quenched with glycine for 5 min, washed in PBS-T (PBS plus 0.1% Triton X-100) and then incubated with primary antibodies (rabbit anti-CK7, 1:1000, Abcam cat#ab68459 RRID: AB_1139824; mouse anti-p53 [DO-1], 1:1000, Santa Cruz Biotechnology cat#sc-126 RRID: AB_628082; rabbit anti-PAX8, 1:100, Abcam cat#ab53490 RRID: AB_2267905; mouse anti-PAR, 1:400, Merck Millipore cat#AM80 RRID: AB_2155072; mouse anti-γH2AX pS139, 1:2000, Merck Millipore cat#05–636 RRID: AB_309864; rabbit anti-pKAP1, 1:500, Bethyl Laboratories cat#A300-767A RRID: AB_669740; rabbit anti-RPA70, 1:500, Abcam cat#ab79398 RRID: AB_1603759; rabbit anti-Rad51, 1:1000, Bio academia cat#70–001 RRID: AB_2177110; sheep anti-CENP-F, 1:1000 [33] in PBS-T or 1% dried skimmed milk (Marvel), after 15 min blocking in 1% milk for PAR staining, for 1 h at room temperature. Coverslips were then washed with PBS-T (× 3) and incubated with the appropriate fluorescent conjugate secondary antibodies (donkey anti-rabbit Cy2, 1:500, cat#711–225-152 RRID: AB_2340612; donkey anti-rabbit Cy3, 1:500, cat#711–165-152 RRID: AB_2307443; donkey anti-mouse Cy2, 1:500, cat#715–225-150 RRID: AB_2340826; donkey anti-mouse Cy3, 1:500, cat#715–165-150 RRID: AB_2340813; donkey anti-sheep Cy3, 1:500, cat#713–165-147; RRID: AB_2315778; all Jackson ImmunoResearch Laboratories) for 30 min at room temperature. Coverslips were then washed with PBS-T (× 3) and DNA was stained for 1 min with 1 μg/ml Hoechst 33258 (Sigma-Aldrich) at room temperature. Coverslips were then washed with PBS-T (× 3) and mounted (90% glycerol, 20 mM Tris, pH 9.2) onto slides. Image acquisition used an Axioskop 2 (Zeiss) microscope fitted with a CoolSNAP HQ camera (Photometrics) using MetaMorph Software (Molecular Devices).

For high-throughput immunofluorescence, cells were processed as above in 96-well plates (PerkinElmer Cell Carrier) with two additional final washes in PBS. For RMG1, wells were coated with 0.01% Poly-L-Lysine. For OCMs, plates were collagen coated (as above) prior to plating. For ovarian cell lines, cells were seeded at 2000–28,000 per well. For OCMs, cells were seeded at 750–6000 cells per well. Images were acquired using Operetta® High Content Imaging System (PerkinElmer) and quantified using Columbus High Content Imaging and Analysis Software (PerkinElmer). Mean fluorescence intensity or foci quantification within the nuclear area (demarcated using Hoechst stain) using Columbus ‘spot finder’ tool, was quantified as a mean value per cell. These were also calculated for secondary antibody-only exposed cells (control). For final values, secondary antibody-only control mean values were subtracted from the stained cell mean values.

### Lentiviral production and transduction

To produce the GFP-H2B cells, AAV293T cells (Agilent Technologies) were plated at 5 × 10^4^ cells per well in a 24-well plate. Media was replenished 1 h before transfection. Cells were transfected with pLVX-based lentiviral plasmids (Takara Bio), modified to express human histone H2B tagged at the N-terminus with GFP (pLVX-myc-EmGFP-H2B) plus psPAX2 and pMD2.G (Addgene) using 16.6 mM CaCl_2_ (Promega) in DMEM supplemented with 10% Hyclone FBS and incubated overnight. Virus was harvested 48 h after transfection, centrifuged and filtered (0.45 μm). Cells were seeded at 2–10 × 10^5^ cells per well in a 12-well plate and diluted lentivirus and 10 μg/ml polybrene (Sigma-Aldrich) added 48 h later. The 12-well plates were centrifuged at 300×g for 2.5 h at 30 °C. 1 mL of culture media was added and the plates incubated overnight. Puromycin (Sigma Aldrich) (2 μg/ml for OVMANA, 1 μg/ml for all other ovarian cancer cell lines and OCMs) was added 48 h after transduction.

### Drug sensitivity assay and cell fate profiling

Ovarian cancer cell lines were seeded, at 500–8000 cells per well and OCMs at 750–6000 cells per well, into a 96-well plate (Greiner Bio-One) 24 h prior to drug treatment. For OCMs, 96-well plates were coated with collagen (as above) prior to plating. To determine half maximal effective concentration (EC_50_), cells expressing GFP-H2B were used and PARGi, PARGi-Me, PARPi, niraparib tosylate and cisplatin were serially diluted from 100 μM–0.381 nM (19 concentrations in total). Paclitaxel was serially diluted from 10 μM–0.0381 nM (19 concentrations in total). Following drug treatment, cells were imaged using an IncuCyte® ZOOM (Essen BioScience) equipped with a 20X objective and maintained at 37 °C in a humidified 5% CO_2_ atmosphere for ovarian cell lines or a humidified 5% CO_2_ and 5% O_2_ atmosphere for OCMs. Nine phase contrast and fluorescence images (for GFP-H2B expressing cells) were collected per well every 4 or 6 h for 120 h to analyse cell proliferation. To determine cell fates, cells were treated with DMSO, PARGi or PARPi, and cells were imaged every 10 min for 120 h.

IncuCyte® ZOOM software was used in real-time to measure green object count in cells expressing GFP-H2B, as a proxy for cell proliferation. Green object count was used to generate dose-response curves in Prism (GraphPad) from which EC_50_ values were calculated. In highly resistant cells, in which EC_50_ could not be determined accurately, the EC_50_ assigned a value of 50 μM (i.e. half the maximum dose tested). To generate cell fate profiles, image sequences were exported in MPEG-4 format and analysed manually to time and annotate cell behaviours [34]. Prism 8 (GraphPad) was used for statistical analysis and presentation.

### Immunoblotting

Ovarian cancer cells lines were treated with DMSO, PARGi or PARPi for 48 h prior to harvesting, or for 2 h with 2 mM Hydroxyurea as a positive control. OCMs were treated with DMSO, PARGi or PARPi for 96 h prior to harvesting, or exposed to 10 Gy of x-ray ionising radiation (IR) using the Faxitron® (Hologic), 4 h prior to harvesting, as a positive control. Proteins were extracted, quantified by Bradford assay, then denatured by boiling in sample buffer (0.35 M Tris pH 6.8, 0.1 g/ml sodium dodecyl sulphate, 93 mg/ml dithiothreitol, 30% glycerol, 50 μg/ml bromophenol blue). Proteins were resolved by SDS-PAGE and electroblotted onto Immobilon-Fl PVDF membrane (Millipore; LI-COR) or Immobilon - P Transfer Membrane (Millipore). For LI-COR imaging, in place of a loading control REVERT total protein stain solution (LI-COR) was used for normalisation: membrane was incubated with REVERT solution for 5 min, followed by washing in 6.7% (v/v) glacial acetic acid in water, 30% (v/v) methanol in water. Before imaging on the Odyssey® CLx Imaging System (Li-COR), the membrane was washed with water. Following imaging, REVERT stain was removed using REVERT reversal solution (0.1 M NaOH, 30% v/v methanol in water). Membranes were then blocked using 5% dried skimmed milk or 5% BSA (for anti-pChk1) diluted in TBS-T (50 mM Tris pH 7.6, 150 mM NaCl, 0.1% Tween-20).

Primary antibodies (mouse anti-Chk1, 1:500, Santa Cruz Biotechnology cat#sc-8408 RRID: AB_627657; rabbit anti-pChk1, 1:750, Cell Signalling cat#2348 RRID: AB_331212; mouse anti-PAR, 1:400, Merck Millipore cat#AM80 RRID: AB_2155072; mouse anti-p53 [DO-1], 1:1000, Santa Cruz Biotechnology cat#sc-126 RRID: AB_628082; sheep anti-Bub3, 1:1000 [A. Holland and S.S. Taylor, unpublished data]; sheep anti-Tao1, 1:1000 [35]) were diluted in 5% dried skimmed milk or 5% BSA (for pChk1) diluted in TBS-T. Membranes were washed in TBS-T (× 3 20 min) and incubated for at least 1 h with the appropriate secondary antibody. For LI-COR, fluorescently-conjugated secondary antibodies (IRDye® 800CW donkey anti-rabbit, 1:5000, cat#925–32,213 RRID: AB_2715510; IRDye® 680RD donkey anti-mouse, Cat#926–68,072 RRID: AB_10953628; both LI-COR) were diluted in 5% dried skimmed milk (Marvel) 0.2% Tween-20 + 0.01% SDS TBS.

For chemiluminescent detection, membranes were incubated with horseradish peroxidase-conjugated antibodies (rabbit anti-sheep IgG (H + L), 1:2000, cat#61–8620 RRID: AB_2533942; goat anti-mouse IgG (H + L), 1:2000, cat#G21040 RRID: AB_2536527; Goat anti-rabbit IgG (H + L), 1:2000, cat#G21234 RRID: AB_2536527; all from Invitrogen) in 5% dried skimmed milk or 5% BSA (for pChk1 antibody) diluted in TBS-T. Membranes were washed TBS-T (× 3 20 min) before secondary horseradish peroxidase-conjugated antibodies were detected using EZ-ECL chemiluminescence reagent (Geneflow) or Luminata Forte Western HRP Substrate (Merck Millipore) and imaged on ChemiDoc Touch Imaging System (BioRad). For LI-COR, membranes were rinsed with TBS and imaged on Odyssey® CLx Imaging System (LI-COR).

### Functional Rad51 assay

OCM.109, OCM.246, OVCAR3 and Kuramochi cells were seeded overnight onto collagen coated (see above) 19 mm coverslips at a cell density of 112,000, 56,000, 36,000 and 36,000 cells per coverslip, respectively. The following day, cells were exposed to 2 Gy of X-ray IR and 1 μM of PARPi or DMSO control for 24 h. Cells were stained for CENPF and Rad51 by the previously stated immunofluorescence protocol. To determine the HR status, the number of CENPF-positive nuclei containing ≥5 Rad51 foci (CENPF+Rad51+) was calculated for 10 separate fields of view, using the Axioskop 2 (Zeiss) microscope, at 40x magnification. A < 2-fold rise in the ratio of Rad51 + CENPF+ cells/CENPF+ cells following IR plus PARPi treatment versus control, was reported as HRD.

### DNA fibre assay

#### Sample preparation

Sub-confluent cells were incubated in the presence of DMSO or PARGi for 48 h, then pulsed with BrdU at 5 μM plus DMSO or PARGi for 20 min. This was followed by 3 washes with warm PBS, pulsing with 200 μM IdU (Sigma-Aldrich) plus DMSO or PARGi for a further 20 min, then washing twice with ice-cold PBS. Following trypsinisation using 0.05% Trypsin-EDTA (Gibco), cells were diluted in ice-cold PBS to give a final concentration of 1–5 × 10^5^ cells/ml and kept on ice.

#### Slide preparation

The cell suspension (2 μl) was then dropped onto microscope slides and dried at room temperature for 5–10 min before mixing with 7 μl of spreading buffer (200 mM Tris-HCl pH 7.5, 50 mM EDTA, and 0.5% SDS), and incubating for a further 5 min. Slides were tilted approximately 5–10° so that the cell suspension runs across the length of the slide. Slides were air dried and fixed in methanol/acetic acid (3:1) for 10 min, air dried and stored at 4 °C.

#### Immunostaining

Prior to immunostaining, slides were washed twice with ddH_2_O for 5 min, 1 × 2.5 M HCl, denatured with 2.5 M HCl for 1 h, rinsed twice with PBS and then washed with blocking solution (PBS with 1% BSA and 0.1% Tween-20) twice for 5 min and then for 1 h. For immuno-labelling all antibodies were dissolved in blocking solution. Slides were then incubated with a rat anti-BrdU antibody (BU1/75 [ICR1], 1:500, Abcam cat# 6326; RRID: AB_305426) to detect BrdU for 1 h under humidified conditions, rinsed with PBS (× 3), fixed for 10 min with 1% formaldehyde, rinsed with PBS (× 3), and quenched with glycine. Slides were then rinsed with PBS (× 3) followed by overnight, 4 °C incubation with mouse anti-BrdU (B44, 1:100, BD Biosciences cat#347580; RRID: AB_400326) to detect IdU. Slides were then washed twice with PBS, 3 times for 5 min in blocking solution, followed by incubation in the appropriate fluorescently-conjugated secondary antibodies diluted in blocking solution (1:500; donkey anti-rat Cy3 cat#712–165-153 RRID: AB_2340667; donkey anti-mouse Cy2 cat#715–225-150 RRID: AB_2340826; all Jackson ImmunoResearch Laboratories) for 1.5 h. Post-incubation, slides were washed 2 x PBS, 3 × 5 min with blocking solution and 2 x PBS. All slides were mounted to coverslips using PBS/Glycerol (1:1).

#### Imaging and quantitation

Images were acquired using an Axioskop 2 (Zeiss) microscope fitted with a CoolSNAP HQ camera (Photometrics) and 2–5 slides analysed per condition. Fibre lengths were quantified using ImageJ software (NIH).

### Genotyping of OCMs

#### *TP53* genotyping by sanger sequencing

RNA was extracted using RNeasy Plus Mini kit (Qiagen) as per manufacturer’s instructions. *TP53* complementary DNA was generated by RT-PCR using Superscript III One Step RT-PCR Platinum Taq HiFi (Thermofisher). PCR products were cloned into a pBluescript SK-vector and transformed into XL1-Blue competent cells. Plasmid DNA was extracted using QIAprep Spin Miniprep Kit (Qiagen) and sequenced using the following primers (5′-CAC CAG CAG CTC CTA CAC CG-3′, 5′-ATG AGC GCT GCT CAG ATA GCG-3′, 5′-CGG CTC ATA GGG CAC CAC C-3′, 5′- TCT TCT TTG GCT GGG GAG AGG-3′). Tumour sequences were aligned using Seqman Pro (DNASTAR).

#### *BRCA1/2* genotyping by next generation sequencing (NGS)

DNA from OCMs was extracted using the cobas® DNA Sample Preparation Kit (Roche). Library enrichment used the GeneRead DNAseq BRCA1 & BRCA2 version 2 kit (Qiagen). 20 ng DNA was amplified in 4 multiplex primer pools. Following PCR-based target enrichment, library preparation and purification followed a custom protocol using AMPure XP beads (Beckman Coulter) for size selection and TruSeq PCR-Free indexes and reagents for indexing (Illumina). The DNA library was then paired-end sequenced on an MiSeq (Illumina) with v2 chemistry (2 × 150 cycles). Bioinformatic analysis used an in-house pipeline, which has been validated to detect low level mosaic calls down to a variant allele fraction of 4% and used a software consensus between VarScan v2.3.6 and DREEP v0.7. Large indel events were assessed using Pindel v0.2.4.t. The NGS assay was able to detect single-nucleotide variants and duplications, deletions and insertions ≤40 base pairs in length, across the whole coding sequence of *BRCA1* and *BRCA2* +/− 15 base pairs beyond each exon-intron junction. The target read depth across all coding exons was a minimum of 350X. All variant calls identified following bioinformatics analysis were independently reviewed within a genome browser. At a variant allele frequency ≥ 4% had a call sensitivity > 95% and specificity > 99% after a manual review.

#### *BRCA1/2* genotyping by multiplex ligation probe amplification (MLPA)

Testing for genomic rearrangements/copy number variation in *BRCA1/2* was performed by MLPA using the MRC Holland probe kits P002-D1 (BRCA1) and P045-C1 (BRCA2). Amplified ligation products were subject to fragment analysis using an ABI 3130xl Genetic Analyser and size called using GeneMapper v2.0 (Applied Biosystems). Copy number status calling was performed using data exported from GeneMapper using custom-developed MLPA spreadsheets that report relative dosage quotient for each probe compared with reference control samples. All MLPA analysis assays were performed in duplicate for confirmation of results.

### scWGS karyotyping

As described previously, [13], single G1 nuclei were isolated by cell sorting then processed for sequencing using a Bravo Automated Liquid Handling Platform (Agilent Technologies) [36, 37]. Samples were sequenced on an Illumina NextSeq 450 at ERIBA (Illumina). Unprocessed sequencing reads were demultiplexed using library-specific barcodes and converted into fastq format using standard Illumina software (bcl2fastq version 1.8.4). Demultiplexed reads were aligned to human reference genome GRCh38 using Bowtie2 (version 2.2.4). Duplicate reads were marked and removed using BamUtil (version 1.0.3.). Aligned sequencing reads were analysed and curated using AneuFinder (version 1.4.0) [36] using 1 Mb bins.

### Characterisation of primary tumours

Formalin-fixed and paraffin-embedded (FFPE) archival tumour blocks were analysed by immunohistochemistry by collecting 4 μm sections on Superfrost charged slides (ThermoScientific). After drying overnight at 37 °C, samples were processed using a Ventana Benchmark immunohistochemistry platform (Roche) with antibodies against p53 (Agilent cat#7001, RRID: AB_2206626, 1:50), CK7 (Agilent cat#7018, RRID: AB_2134589, 1:250), PAX8 (Roche cat#760–4618, 1:100). Heat induced epitope retrieval was performed using CC1 (Roche), incubating samples at 95 °C for 36, 52, and 40 min for p53, CK7 and PAX8, respectively. Antibodies were incubated at 37 °C for 32, 40 and 32 min for p53, CK7 and PAX8, respectively. p53 and CK7 were detected using Ultraview universal DAB kit (Roche), while PAX8 was detected using Optiview universal DAB kit (Roche), all as per manufacturer’s instructions. Sections were counterstained using Haematoxylin II (Roche) for 12 min and bluing reagent (Roche) for 8 min. Slides were imaged using the EVOS FL Auto 2 Imaging System (Invitrogen), using a × 10 or × 40 objective lens under bright field, and processed using Adobe Photoshop.

### NanoString of ovarian cancer cell lines

RNA was extracted using RNeasy Plus Mini kit (Qiagen) as per manufacturer’s instructions. 100 ng RNA was provided at concentration of 4 ng/μl to the Genomic Technologies Core Facility at University of Manchester for analysis using the NanoString nCounter Analysis System. Twenty-five replication genes, 25 mitotic genes, 25 apoptotic genes (Fig. 4B) and 4 reference genes as normalisation controls appropriate for use in ovarian cancer (*RPLP0*, *PPIA*, *IPO8*, *TBP*) were analysed using nSolver™ Analysis Software 4.0., which generated normalised transcript counts.

### Analysis of mitotic, DNA replication and apoptotic gene expression in RNAseq datasets from the CCLE and Klijn et al., (2015)

Previous analysis utilised gene expression microarray profiling from the CCLE to identify ovarian cancer cell lines with low expression of DNA replication genes [26, 38]. Here, we updated this analysis to utilise the more recent RNAseq performed by the CCLE [39] (Fig. 4C). Briefly, read counts were obtained from the Broad Institute data portal (https://portals.broadinstitute.org/ccle). The R package DESeq2 (v1.26.0) was used to normalise and apply a variance stabilising transformation to the assembled read count matrix, followed by z-score transformation.

We additionally utilised read counts for the panel of 10 ovarian cancer cell lines of the same DNA replication, mitotic and apoptotic gene lists from a pan-cancer cell line RNAseq study [40] (GEO accession number GSE40788). Raw read counts were treated as described previously. This revealed higher expression of these genes in OV56 cells than indicated in the CCLE data set, in agreement with our NanoString data (Fig. S4A).

### RNA-sequencing and analysis

RNA was extracted using RNeasy Plus Mini kit (Qiagen), quantified using a Qubit fluorometer (Life Technologies) and quality/integrity assessed using a 2200 TapeStation (Agilent Technologies). Sequencing libraries were then generated using the TruSeq® Stranded mRNA assay (Illumina, Inc.) according to the manufacturer’s protocol. Adapter indices were used to multiplex libraries, which were pooled and paired-end sequenced on an Illumina HiSeq4000 instrument. The output data was demultiplexed (allowing one mismatch) and BCL-to-Fastq conversion performed using Illumina’s bcl2fastq software. Overlapping paired reads were merged using BBMerge, and trimming and filtering was done using BBDuk, both from BBMap v36.32. The filtered reads were mapped to the human reference sequence analysis set (hg38/Dec. 2013/GRCh38) from the UCSC browser, using STAR v2.7.2b [41]. The genome index was created using the comprehensive Gencode v32 gene annotation. The number of reads per gene were counted using ‘--quantMode GeneCounts’ within the STAR command.

Of the 32 OCMs profiled for PARGi and PARPi sensitivity, 29 have been analysed by RNAseq across 6 sequencing runs [13, 30, 42]. Data analyses in R was performed using v3.6.2 and Bioconductor v3.10. The DESeq2 (v1.26.0) package was used to apply a variance stabilising transformation to the assembled read count matrix [43]. A z-score transformation was then applied and OCMs ranked by the sum of z-scores for 25 mitotic, DNA replication and apoptotic gene (lists as described above). Heatmaps were created using ComplexHeatmap v2.6.2 [44]. PARGi-sensitivity was defined using the colony formation assay data in Fig. S6B, whereby treated OCMs with < 50%, ≥50–< 90%, and ≥ 90% colony area of control are defined as PARGi-sensitive, partially PARGi-sensitive, and PARGi-resistant, respectively.

### Quantification and statistical analysis

Statistical analysis was carried out using GraphPad Prism 8 Software. *P* values were designated as follows: * < 0.05, ** < 0.01, *** < 0.001, **** < 0.0001, ns *p* > 0.05. Details of statistical analyses are described in the figure legends.

## Results

### Identification of PARGi sensitive and resistant ovarian cancer cell lines

**Fig. 1:**
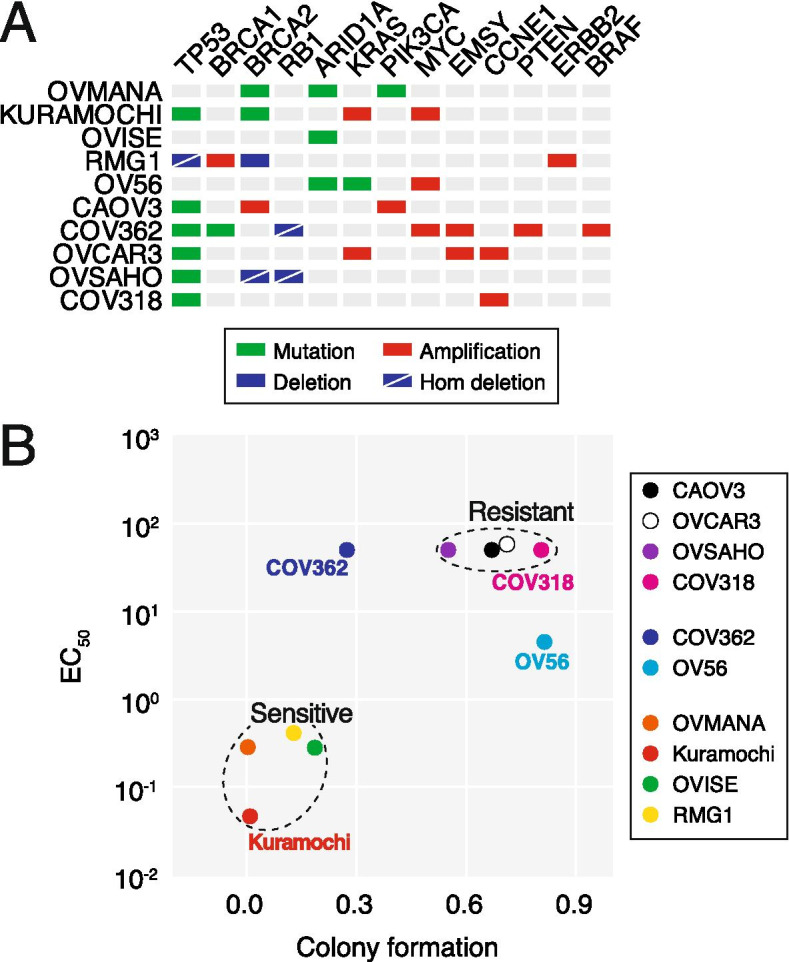
Identification of PARGi-sensitive and -resistant ovarian cancer cell lines. **A** Mutational profile of ovarian cancer cell line panel [45]. **B** XY plot showing correlation between EC_50_ and colony formation with PARGi treatment. Values derived from ≥2 experiments (EC_50_) or ≥ 3 (colony formation). See also Fig. S1

To analyse the molecular mechanisms underlying PARGi sensitivity, we assembled a panel of ten ovarian cancer cell lines: OVMANA, Kuramochi, OVISE, RMG1, OV56, CAOV3, COV362, OVCAR3, OVSAHO and COV318 (Fig. 1A). While six of these cell lines have genetic hallmarks of HGSOC, in particular *TP53* mutations (Fig. 1A), OVMANA, OVISE, RMG1 and OV56 are more representative of ovarian clear cell carcinoma [30, 45]. Nevertheless, we reasoned that analysing a panel of epithelial ovarian cancer cell lines with potential for PARGi sensitivity would provide insight into the intrinsic vulnerabilities responsible for PARGi sensitivity, and in particular provide insight into whether PARGi sensitivity was occurring via a common mechanism.

Previously, we showed that Kuramochi, OVMANA and RMG1 are PARGi sensitive in long-term colony formation assays, while COV362, CAOV3, OVCAR3, OVSAHO and COV318 were resistant in a short-term proliferation assay. While OV56 was identified as resistant by colony formation assay, OVISE was also classified as resistant because PARGi treatment failed to induce pan-nuclear γH2AX [26]. Note however that in our previous study, analysis of OVISE was challenging due to technical issues, which we have now overcome. To systematically compare PARGi sensitivity, all ten cell lines were now analysed in long-term colony formation assays following exposure to 1 μM PARGi for 24, 48, 72 h or continuously, and colony area quantitated. Examination of continuously treated cells confirmed that Kuramochi, OVMANA and RMG1 are indeed PARGi sensitive (Fig. S1A, B). However, in contrast to our previous report, this latest analysis shows that OVISE is also PARGi sensitive. Outgrowth of OVMANA, Kuramochi and OVISE was also inhibited following drug exposure for 48 and 72 h, while RMG1 was not (Fig. S1C). In line with our previous study, growth of OV56, CAOV3, OVCAR3, OVSAHO and COV318 was not markedly suppressed even in the continuous presence of PARGi, while COV362 growth was partially suppressed (Fig. S1B). In addition, two out of four PARGi-sensitive cell lines were resistant to PARPi (Kuramochi and RMG1), while four out of six PARGi-resistant lines were sensitive to PARPi (Fig. S1A, G, H), confirming differential sensitivity in some cases, despite both inhibitors targeting PARylation.

To independently analyse PARGi sensitivity, each cell line harbouring a GFP-tagged histone was analysed by time-lapse microscopy, over a 120-h period, in the presence of increasing concentrations of PARGi (Fig. S1D). Proliferation curves were then analysed to determine nuclear doubling rates and in turn calculate EC_50_ values (Fig. S1E). COV318, OVSAHO, OVCAR3, COV362 and CAOV3 were largely unaffected even at very high concentrations of PARGi, yielding high or indeterminant EC_50_ values (Fig. S1F). By contrast proliferation of RMG1, OVMANA and OVISE was strongly inhibited, yielding EC_50_ values in the micromolar range (Fig. S1F). Consistent with our previous analysis, Kuramochi were particularly sensitive with an EC_50_ value of 46 nM.

To integrate these two data sets, we plotted EC_50_ values from the proliferation assays against colony area in the continuous presence of PARGi (Fig. 1B). This clearly highlights OVMANA, Kuramochi, OVISE and RMG1 as PARGi-sensitive, and OVSAHO, CAOV3, OVCAR3 and COV318 as PARGi-resistant. COV362 and OV56 are more ambiguous. While OV56 yields an EC_50_ of ~ 4.5 μM, these cells clearly form colonies in 1 μM of PARGi and closer inspection reveals that these colonies are less dense than controls (Fig. S1A), suggesting that although PARGi slows OV56 proliferation it does not block it enough to prevent outgrowth. With an indeterminant EC_50_, COV362 appears PARGi-resistant, and closer inspection reveals large colonies indicating outgrowth (Fig. S1A). Thus, we conclude that both COV362 and OV56 are PARGi resistant. In summary, taking together the long-term colony formation assay and the short-term nuclear proliferation assay, we conclude that OVMANA, Kuramochi, OVISE and RMG1 are PARGi-sensitive while OV56, CAOV3, COV362, OVCAR3, OVSAHO and COV318 are PARGi-resistant.

### PARGi stabilises PAR chains in both sensitive and resistant cell lines

A possible explanation for PARGi resistance could simply be that the PARGi fails to engage with its target in resistant cells, for example due to drug efflux mechanisms. Therefore, we sought to determine whether PARGi was indeed inhibiting PARG activity in all ten cell lines, by measuring the accumulation of PAR chains. Analysis of single cells by immunofluorescence microscopy showed that in all cell lines, exposure to PARGi increased the intensity of nuclear PAR staining (Fig. S2A–C). Importantly, increased PAR staining was blocked by co-treatment with an equimolar concentration of PARPi, indicating that accumulation of PAR chains was dependent on PARP activity (Fig. S2A–C). Immunoblotting also revealed increased PAR levels in PARGi-treated versus untreated cells, with accumulation of higher molecular weight PAR species indicating stabilisation of PAR chains (Fig. S2D–F). However, the very high molecular weight PAR species did not accumulate in OV56 or Kuramochi, and indeed, there is inter-line variation in PAR-chain dynamics (Fig. S2E). For OV56, although increased PAR staining with PARGi treatment was seen by microscopy, the increase was minimal by immunoblotting. This disparity is perhaps due to differences in assay sensitivity and/or arises from observing individual cells versus the whole population. Nevertheless, the two approaches support that PARGi resistance is not explained by a failure to stabilise PAR chains, but rather by an ability to tolerate the presence of stabilised PAR chains.

### PARGi sensitivity is accompanied by markers of replication stress

**Fig. 2:**
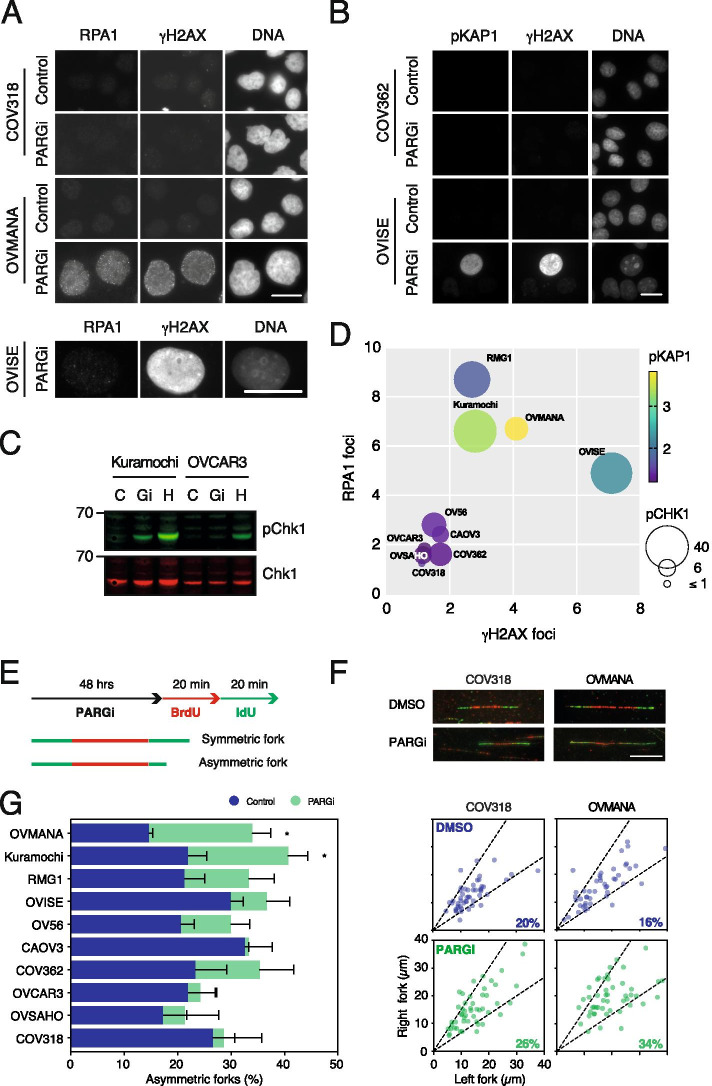
PARGi sensitivity is accompanied by markers of replication stress and the DNA damage response. **A** Representative images of RPA1 and γH2AX foci in response to 48 h of 1 μM PARGi treatment or DMSO (Control) in PARGi-resistant (COV318) and PARGi-sensitive (OVMANA) cell lines (upper). RPA1 and pan-nuclear γH2AX staining also shown in PARGi-sensitive OVISE cells following 72 h PARGi treatment (lower). Scale bars: 20 μm. **B** Representative images of pan-nuclear pKAP1 and γH2AX staining in PARGi-resistant (COV362) and PARGi-sensitive (OVISE) cell lines following 72 h of 1 μM PARGi treatment. Scale bar: 20 μm. **C** Representative immunoblot Li-COR image for PARGi-resistant (OVCAR3) and PARGi-sensitive (Kuramochi) cell lines; cells were treated for 48 h with 1 μM PARGi (Gi) or DMSO as a negative control (C), or for 2 h with 2 mM hydroxyurea (H) as a positive control. **D** Bubble plot showing fold-change in RPA1 foci, γH2AX foci, pan-nuclear pKAP1 and Chk1 phosphorylation in response to PARGi treatment. Mean of *n* ≥ 3 biological replicates for each parameter quantified using single-cell immunofluorescence microscopy. Bubble size represents pChk1, bubble colour represents pKAP1. (**E**) Schematic of DNA fibre experimental strategy. (**F**) Exemplar fibres in PARGi-resistant (COV318) and PARGi-sensitive (OVMANA) cell lines. Scale bar: 10 μm. (**G**) Mean % asymmetric forks are shown on the left. The exemplar graphs on the right show correlation between R and L fork lengths. Dotted lines represent 33% cut-off, beyond which forks are considered asymmetric (% asymmetry for replicate shown in respective graph bottom right corner). Statistics: Mean of 3 biological replicates, with ≥50 forks measured per cell line, per condition. 2-way ANOVA with Sidak post-hoc test, selected comparisons were between PARGi treated values and DMSO control within each cell line. Error bars represent SEM. * *p* < 0.05. See also Fig. S3

Having established that PAR chains accumulate with PARGi treatment in all ten cell lines, we asked whether PARGi sensitivity occurs via a similar mechanism, in particular via DNA replication catastrophe. Previously, we showed that PARGi-sensitive Kuramochi cells displayed features consistent with persistent replication stress, DNA damage and eventually replication catastrophe upon prolonged exposure to PARGi [26]. If this phenomenon is a common cause of PARGi sensitivity, we reasoned that these features should also manifest in other sensitive lines, but not resistant lines. To test this, we measured accumulation of three well recognised markers of replication stress, namely γH2AX foci, RPA foci, and nuclear phospho-KAP1 [29, 46, 47] using high-throughput immunofluorescence microscopy (Fig. 2A, B and S3A, B). After 48 h of exposure to PARGi, γH2AX foci were substantially and significantly increased in the sensitive lines OVISE, Kuramochi and OVMANA, but not in the six resistant cell lines (Fig. 2A and S3A, B). By 72 h, cells with pan-nuclear γH2AX staining also became apparent (Fig. 2A, B). Induction of RPA1 foci and nuclear phospho-KAP1 showed a similar trend (Fig. 2A, B and S3A), confirming a correlation between PARGi sensitivity and markers of replication stress. To independently analyse replication stress, we analysed cell populations by immunoblotting to measure phosphorylation of Chk1, normalised to total Chk1, and using the ribonucleotide reductase inhibitor hydroxyurea (HU) as a positive control (Fig. 2C). This was initially done by chemiluminescent western blot (Fig. S3C), but subsequently repeated using LI-COR system to enable quantification (Fig. 2C, S3D). Because Chk1 phosphorylation in response to HU exhibited substantial variation between the cell lines, we expressed the phosho-Chk1/total-Chk1 ratio as a percentage of the HU-induced maximum. This shows that PARGi induced strong Chk1 phosphorylation responses in three of the sensitive lines RMG1, OVISE and Kuramochi, but not in the resistant lines (Fig. S3C, D), further suggesting a correlation between PARGi sensitivity and replication stress.

We did notice a few exceptions to this overall trend. While RMG1 cells are sensitive and showed a substantial fold change in RPA foci and strongly induced phosho-Chk1, they showed only modest increases in γH2AX foci and phosphorylated-KAP1 (Fig. S3A, D). Also, while CAOV3 cells are PARGi resistant, they showed induction of RPA1 foci, but not the other markers, suggesting this level of replication stress is tolerated sufficiently to allow growth (Fig. S3A). Indeed, it has previously been reported that PARG is required for recovery from persistent, but not short-term replication stress [48]. These exceptions highlight the limitations of relying on a single marker of replication stress. Therefore, we integrated these four datasets by plotting the fold changes of γH2AX foci versus RPA foci, phospho-KAP1 and phospho-Chk1 (Fig. 2D). This analysis shows a clear demarcation of sensitive and resistant cell lines, with OVISE, Kuramochi, RMG1 and OVMANA segregated from the six resistant cell lines. Thus, in toto, these observations demonstrate that PARGi sensitivity does reflect a common mechanism in ovarian cancer cell lines, namely persistent replication stress leading to replication catastrophe.

### PARGi induces replication fork asymmetry in sensitive cell lines

We previously showed that inhibiting PARG causes DNA replication fork asymmetry in sensitive Kuramochi cells, but not resistant OVCAR3 cells [26], reflecting the role of PARG in restarting stalled replication forks. Having established that all four sensitive lines demonstrated features of replication stress when exposed to PARGi, we asked whether this was accompanied by persistent fork stalling, which we assessed using fork asymmetry assays [49]. Following a 48-h exposure to PARGi, cells were pulsed with BrdU to label active DNA replication forks, then chased with IdU to measure the speed of left and right cognate forks (Fig. 2E, F). The three cell lines in the panel with the highest fold-change (1.7–2.3 fold) in fork asymmetry following PARGi-treatment are PARGi-sensitive (OVMANA, Kuramochi, RMG1; Fig. 2G); whereas COV362 cells, despite a fold-change of 1.5, are PARGi-resistant. Intriguingly, these four cell lines possess *BRCA1/2* mutations or deletions (Fig. 1A); accordingly, fork asymmetry following PARGi-treatment could be due to an absence of both PARG- and HR-mediated fork restart [27]. However, PARGi-resistant OVSAHO has a homologous deletion in *BRCA2* and does not show increased fork asymmetry following treatment. Furthermore, asymmetry only increased marginally in PARGi-sensitive OVISE, which is *BRCA1/2* wild-type, although these cells have intrinsically high levels of asymmetry, possibly indicating an existing fork-restart vulnerability in these cells (Figs. 1A and 2G). The level of fork asymmetry conferring PARGi-sensitivity may therefore differ between cell lines, and is likely modulated by other factors in addition to HR status, such as the ability to utilise dormant replication origins. Indeed, there is variation in basal level of fork asymmetry across the panel (Fig. 2G). Nevertheless, we conclude therefore that PARGi sensitivity generally is accompanied by an induction of DNA replication fork asymmetry, indicating persistent fork stalling, further supporting the notion that PARGi sensitivity is due to persistent replication stress.

### PARGi suppresses mitotic entry in sensitive cell lines

**Fig. 3:**
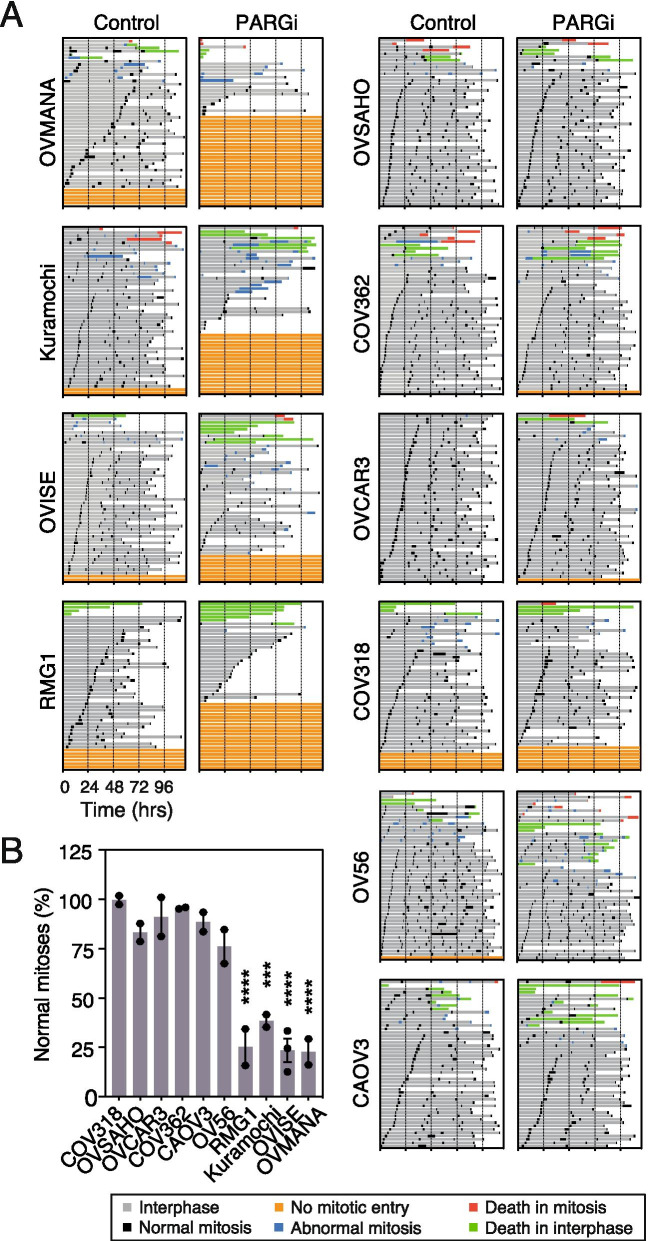
PARGi suppresses mitotic entry in sensitive cell lines. **A** Cell fate profiling, showing cell behaviour over 115 h treatment with 1 μM PARGi or DMSO (Control). Each horizontal line represents a single cell, with the colours indicating cell behaviour. Following mitosis, one daughter cell was chosen at random to continue the analysis. No mitotic entry does not include death in interphase where mitosis does not take place. Abnormal mitosis includes cell division abnormalities such as slippage (where cells enter mitosis, but exit without division), fusion (where daughter cells appear to separate but subsequently join back together), tripolar cell divisions and division of binuclear cells. **B** The % of total normal mitoses in PARGi-treated compared with DMSO controls. Mean of ≥2 biological replicates. Statistics: total normal mitoses in DMSO-treated compared with PARGi-treated cells was compared by 2-way ANOVA with Sidak post-hoc test. Error bars represent SEM. ***p < 0.001, ****p < 0.0001

Persistent replication stress activates intra-S and G_2_/M checkpoints, thereby blocking cell cycle progression, and in particular inhibiting entry into mitosis. Indeed, we previously showed that a substantial fraction of PARGi-treated Kuramochi cells underwent a Wee1-dependent G_2_ arrest, and that those cells that did progress through mitosis often did so abnormally, and subsequently died [26]. To determine whether a similar cell fate was shared by other PARGi-sensitive lines, we analysed the panel of ten cell lines using time-lapse microscopy for 115 h and generated cell fate profiles [34]. In the absence of PARGi, the vast majority of cells in each line underwent multiple successful divisions and were alive at the end of the experiment (Fig. 3A). Significantly, PARGi treatment of the sensitive cell lines resulted in an increased proportion of cells that did not enter mitosis, with 48, 32 and 51% of OVMANA, Kuramochi and RMG1, respectively, blocking in interphase (Fig. 3A). This phenotype was not observed in the resistant cell lines where the majority of cells continued to complete multiple cell divisions when treated with PARGi.

Interestingly, in the four sensitive cell lines, a small proportion of cells did not enter mitosis in the absence of PARGi, consistent with these cells exhibiting an underlying vulnerability that is markedly exacerbated by inhibition of PARG. Of the resistant lines, a small proportion of COV318 cells also did not enter mitosis in the absence of PARGi, but in contrast to the sensitive lines, this did not increase substantially upon exposure to PARGi.

In most of the cultures analysed, a fraction of cells underwent abnormal mitoses and/or cell death and this phenotype was exacerbated by PARGi in sensitive but not resistant lines. In particular, while only 14% of PARGi-treated OVISE cells blocked in interphase, the number of cells undergoing abnormal mitoses and/or cell death increased from 17 to 46% (Fig. 3A). Although the analyses above indicate that OV56 is PARGi-resistant, the cell fate profiling indicates that drug exposure increases the number of abnormal mitoses from 23 to 35% (Fig. 3A), possibly reflecting a synthetic effect with the prolonged cell culture and/or imaging conditions.

To quantitate these PARGi effects on mitotic potential, we counted the number of productive cell divisions in control and drug-treated populations. In sensitive cell lines, PARGi dramatically reduced the number of normal mitoses, by an average of 73%, compared with a minor reduction (11% on average) in resistant lines (Fig. 3B). Thus, considering both the cell fate profiles and the quantitation of successful divisions, we conclude that sensitivity to PARGi is accompanied by a dramatic loss of mitotic potential, in particular a pre-mitotic block, consistent with activation of intra-S and G_2_/M checkpoints due to persistent replication stress.

### PARGi sensitivity correlates with replication stress

**Fig. 4:**
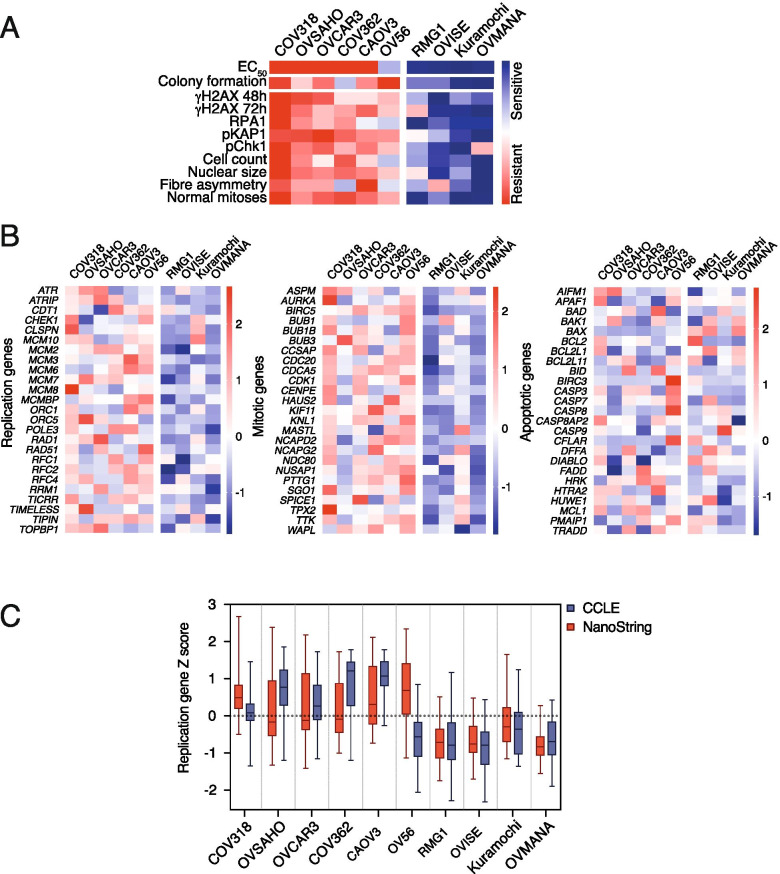
PARGi-sensitive cell lines have lower expression of DNA replication genes. **A** PARGi-sensitivity assay results summarised as a heatmap. Assay results (except EC_50_ values) were scaled from 0 to 1, averaged, and cell lines rank ordered accordingly. γH2AX at 48 h is based on nuclear foci quantification. γH2AX at 72 h is based on nuclear intensity. **B** NanoString DNA replication, mitotic and apoptotic gene Z-scores of cell lines represented by heatmaps where red = high expression; white = average expression and blue = low expression. **C** Box-whisker plots (min–max) for DNA replication genes Z-scores shown for NanoString compared with RNA-sequencing (RNAseq) data from the CCLE [39]. See also Fig. S4

The analyses described above show that markers of replication stress (Fig. 2A–D), fork asymmetry (Fig. 2E–F) and a pre-mitotic block (Fig. 3), associated with increased nuclear size and reduced cell count (data not shown), also appear to correlate with PARGi sensitivity. To confirm this, we integrated the various datasets by scaling each parameter so that the most resistant and sensitive lines scored 1 and 0 respectively (Fig. 4A). These were then averaged to yield an overall sensitivity score and rank ordered. Importantly, the various cell biological parameters clearly align with sensitivity as defined by the colony formation and short-term proliferation assays, with COV318, OVSAHO, OVCAR3 and COV362 ranking as the most resistant lines, and OVMANA, Kuramochi and OVISE the most sensitive. Of the sensitive lines, RMG1 ranks as the least sensitive and indeed, as noted above, this line requires continuous exposure for fully penetrant inhibition (Fig. S1A–C). Of the resistant lines, OV56 is the least resistant and, again as noted above, although colonies form in the presence of PARGi, they are less dense than control colonies (Fig. S1A, B). CAOV3 are also at the more ‘sensitive’ end of the resistant spectrum and interestingly, these cells do display increased RPA foci when exposed to PARGi (Fig. S3A), suggesting that while PARGi does induce replication stress, it is resolved sufficiently to allow efficient recovery. Nevertheless, this integrated analysis confirms that PARGi sensitivity does indeed correlate with markers of replication stress in ovarian cancer cell lines, suggesting a common underlying mechanism. Moreover, because PAR chains are stabilised in all cell lines analysed (Fig. S2C, D), an important corollary is that resistant cell lines can efficiently complete DNA replication despite the presence of stabilised PAR chains.

### PARGi sensitivity correlates with lower expression of DNA replication genes in cell lines

The strong correlation between markers of replication stress and PARGi sensitivity supports our previous conclusion that sensitive cell lines harbour an underlying DNA replication vulnerability that makes them particularly dependent on PARG activity to re-start stalled replication forks [26]. While the nature of this vulnerability remains to be determined, when we previously interrogated CCLE microarray data, we noted that in addition to Kuramochi, the expression levels of a number of DNA replication genes was lower in RMG1, OV56, OVMANA and OVISE cell lines compared with resistant lines [26]. Of these four, we previously showed that RMG1 and OVMANA are PARGi-sensitive, and here we demonstrate that OVISE is also sensitive. However, while OV56 is the most ‘sensitive’ of the resistant lines, it is resistant despite apparent low expression levels of DNA replication genes. To address this anomaly, we set out to independently validate the expression levels of 25 DNA replication genes using a custom NanoString CodeSet (Fig. 4B) and compare the overall expression of these genes with more recent RNA-sequencing (RNAseq) from the CCLE project [39] (Fig. 4C). NanoString analysis confirmed that expression of the 25 DNA replication genes is lower in PARGi-sensitive than -resistant lines, and for eight cell lines the CCLE and NanoString data significantly correlated (Fig. S4A). While the correlation between NanoString and CCLE for RMG1 is not significant, both find that this PARGi-sensitive line has lower expression of these genes than resistant lines. However, in contrast with the CCLE data, we find that OV56 have relatively high expression of the DNA replication genes in line with other resistant lines (Fig. 4C, S4A). Interestingly, while the more recent RNAseq data from the CCLE project is consistent with their previous microarray data [38, 39], an independent RNAseq study of cancer cell lines also indicates higher expression of DNA replication genes in OV56 [40]. Indeed, the OV56 data from Klijn et al., significantly correlated with our NanoString expression data, but not the CCLE data (Fig. S4A). Aside from the anomaly of OV56, two independent RNAseq datasets [39, 40] and our NanoString-based analysis therefore indicate that cell lines that are sensitive to PARGi tend to have lower expression levels of a number of DNA replication genes.

If PARGi-sensitivity specifically correlates with DNA replication gene expression, we expected the expression level of genes involved in other processes not to be associated with sensitivity. To test this, we expanded our analysis to include 25 mitotic genes and 25 apoptotic genes, based on NanoString CodeSets we used previously [50]. While apoptotic gene expression was variable amongst both sensitive and resistant cell lines, expression of the mitotic genes was lower in PARGi-sensitive versus -resistant lines (Fig. 4B, S4A). However, we also observed a striking correlation between expression of replication and mitotic genes in our cell line panel (NanoString: R^2^ = 0.87, *p* < 0.0001 [not shown]; CCLE: R^2^ = 0.96, p < 0.0001) (Fig. S4B). Replication and mitotic gene expression also correlated in a wider cohort of 747 cancer cell lines within the CCLE database (R^2^ = 0.72, p < 0.0001), but this was not observed for replication versus apoptotic genes (R^2^ = 0.081) (Fig. S4B). Therefore, while the NanoString analysis builds on our previous analysis [26], showing association between low expression of DNA replication genes and PARGi sensitivity, we cannot rule out a wider cell cycle control gene expression phenotype.

### Differential sensitivity to PARPi and PARGi

As PARG acts as a direct counterbalance to PARP-1/2, and further therapeutic strategies are needed for disease with inherent or acquired PARPi resistance, we wondered whether PARGi-sensitive cell lines showed differential sensitivity to PARPi, and vice versa. In terms of PARPi sensitivity, using a short-term confluence assay, we previously showed that OVSAHO, COV318, COV362 and CAOV3 were PARPi-resistant, whereas OVCAR3 was sensitive [26]. Because long-term target engagement can be required to observe synthetic lethality between cells with a *BRCA1/2* mutation and Olaparib [28], we re-visited this and compared PARPi and PARGi sensitivity in long-term colony formation assays with continuous drug exposure. Of these four lines, with the exception of COV318, all showed a reduction in outgrowth in response to PARPi (Fig. S1G). By contrast, and in agreement with our previous report, OV56, Kuramochi and RMG1 were relatively resistant to PARPi. Notably, OVISE and OVMANA were sensitive to both PARPi and PARGi. Thus, we conclude that of the ten ovarian cancer cell lines analysed here, four are PARPi-sensitive and PARGi-resistant; two are PARGi-sensitive and PARPi-resistant; two are sensitive to both treatments and two are resistant to both (Fig. S1H). This implies that sensitivity to PARGi and PARPi is neither mutually exclusive nor overlapping, indicating that the determents of sensitivity are context dependent. In this regard OV56 is an interesting case – while categorised as PARGi-resistant, it is the least ‘resistant’ of the resistant lines and PARGi exposure is clearly not completely inconsequential: the short-term proliferation assay yields high EC_50_ values for PARPi and PARGi (Fig. S1F, G), and the colonies are less dense than controls for the PARGi-treated culture (Fig. S1A). In contrast, in response to PARPi, it forms robust colonies (Fig. S1A). Thus, despite the intimate relationship between PARP and PARG activities, and despite the complexities described above, a differential sensitivity clearly manifests across multiple cell lines, indicating that PARG inhibitors may offer an alternative therapeutic option to target a subset of ovarian cancers, such as HRP tumours that are less likely to respond to PARPi.

### Identification of PARGi-sensitive patient-derived ovarian cancer models

**Fig. 5:**
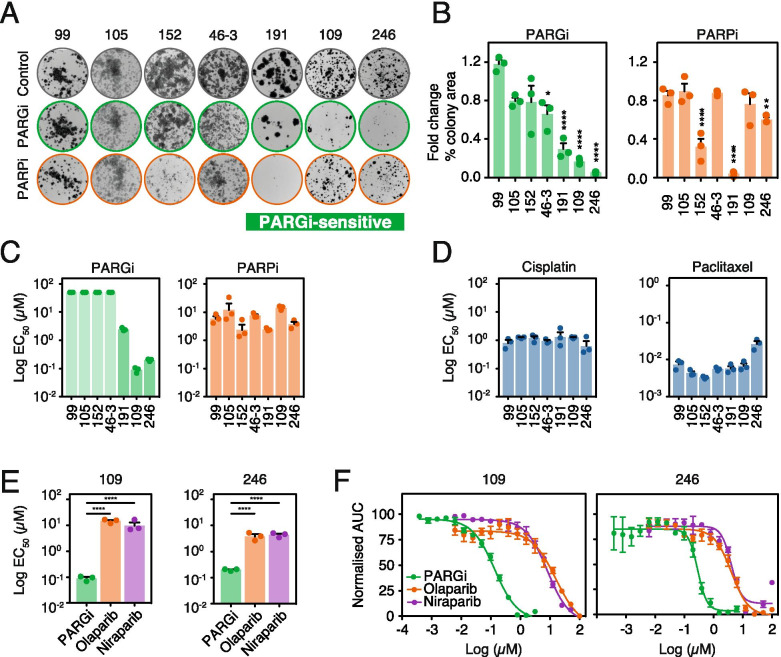
Drug sensitivity of OCMs. **A** Colony formation following 96 h of treatment with 1 μM PARGi or 1 μM PARPi or DMSO (Control). Representative images of 3 biological replicates. **B** Quantification of ca from (A) normalised to DMSO-treated cells (Control) and represented as fold-change. Mean of 3 biological replicates. **C** Proliferative Log EC_50_ values for PARGi and PARPi (Olaparib) in panel of seven OCMs. Means of 3 biological replicates. **D** Proliferative Log EC_50_ values for cisplatin and paclitaxel in panel of seven OCMs. Means of 3 biological replicates. **E** Proliferative Log EC_50_ values for PARGi, Olaparib (PARPi) and Niraparib for PARGi-sensitive OCMs (109 and 246). Mean of 3 biological replicates. **F** Dose-response curves for PARGi, Olaparib (PARPi) and Niraparib for PARGi-sensitive OCMs (109 and 246). Mean of 3 biological replicates. PRISM could not accurately calculate EC_50_ for PARGi-resistant cells, therefore for resistant OCMs the EC_50_ was approximated as 50 μM (half the maximal concentration tested). Error bars represent SEM. Statistics: 1-way ANOVA with Dunnett’s multiple comparisons test of PARGi versus Olaparib (*P* < 0.0001) and PARGi versus niraparib (P < 0.0001). * p < 0.05, ** *p* < 0.01, ****p < 0.0001. See also Supplementary Table 3, and Figs. S6 and S7

Having demonstrated that PARGi sensitivity in established ovarian cancer cell lines arises due to replication catastrophe, we determined the impact of PARGi on a panel of 32 patient-derived OCMs from a living biobank focused on women treated at The Christie Hospital [13]. In contrast to established cell lines, these OCMs are clinically annotated and available for analysis at low passage. Seven OCMs were described previously [13], and with 25 additional OCMs described here (Supplementary Table 1). Importantly, these new OCMs also displayed the features of HGSOC, namely nuclear atypia, universal expression of CK7 and PAX8, aberrant p53 expression, *TP53* and *BRCA1/2* mutations, and extensive chromosomal instability (Fig. S5; Supplementary Table 1). To screen for PARGi sensitivity, the panel was exposed to 1 μM drug for 96 h and colony area (ca) measured after ≥2 weeks (Fig. S6A). Of the 32, 15 were largely unaffected (ca ≥ 90%), 10 partially affected (50% ≤ ca < 90%) and seven substantially affected (ca < 50%) (Fig. S6B). Of these seven sensitive OCMs, 191, 109 and 246 were then further analysed in parallel with four resistant OCMs, namely 99, 105, 152 and 46–3 (Fig. 5A, B). First, we confirmed relative sensitivity, both in a colony formation assay in response to continuous PARGi exposure (Fig. S6D, E), and in a time-lapse microscopy-based short-term proliferation assay tracking GFP-tagged nuclei (Fig. 5C). Note that an N-methylated analogue of PARGi with minimal activity in vitro [28] was inactive against OCMs 191, 109 and 246 (Fig. S7), consistent with sensitivity due to on-target inhibition of PARG. Interestingly, there was no obvious correlation with cisplatin or paclitaxel sensitivity (Fig. 5D), and while OCMs 109 and 246 were resistant to the PARP inhibitors Olaparib and Niraparib (Fig. 5E, F), OCM.191 was PARPi-sensitive (Fig. S6D, E). Indeed, the screen identified eight PARPi-sensitive OCMs, five of which were PARGi-resistant, while 191, 80–2, 59–3 were PARGi-sensitive (Fig. S6A–C). Thus, in summary, out of 32 patient-derived OCMs, we identified seven sensitive to the PARGi and eight sensitive to PARPi, including three sensitive to both.

### PARG inhibition also induces replication catastrophe in sensitive OCMs

**Fig. 6:**
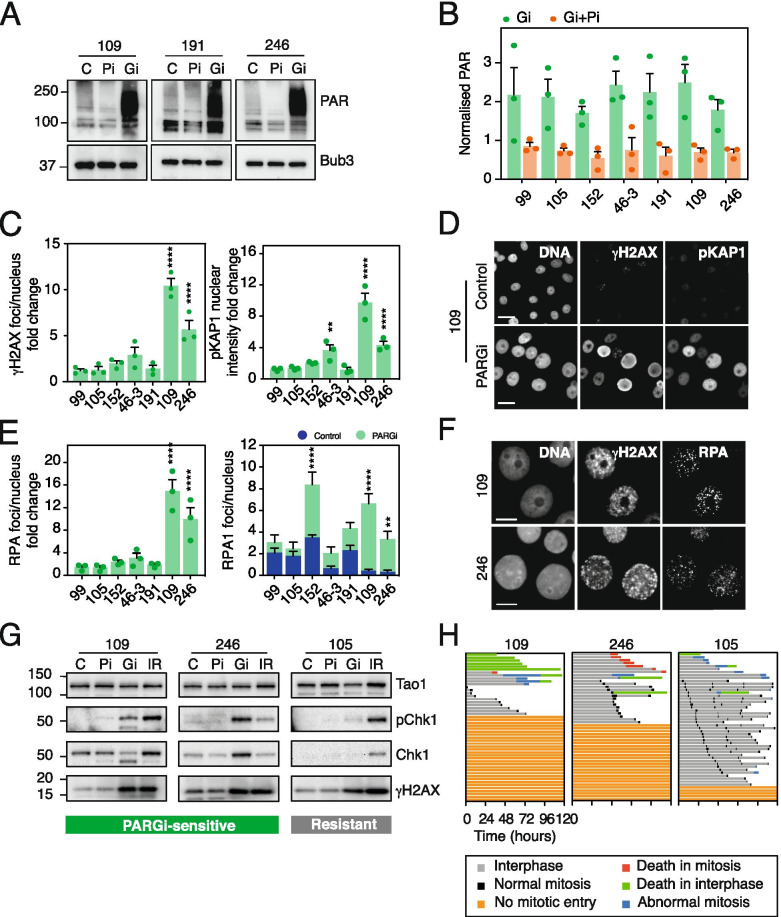
Mechanism of PARGi sensitivity in OCMs. **A** Representative immunoblots showing PAR chain formation in OCMs following 96 h of treatment with DMSO (C), 1 μM PARPi (Pi) or 1 μM PARGi (Gi). **B** Quantification of PAR staining in response to PARGi (Gi) or PARGi co-treated with PARPi (Gi + Pi) using single-cell immunofluorescence microscopy, normalised to DMSO-treated cells (Control). Mean of 3 biological replicates. Error bars represent SEM. **C** Quantification of γH2AX foci per nucleus and nuclear pKAP1 intensity after 72 h of 1 μM PARGi using single-cell immunofluorescence microscopy, normalised to DMSO-treated cells (Control) and represented as fold change. Mean of 3 biological replicates. Error bars represent SEM. Statistics: 2-way ANOVA with Dunnett’s multiple comparisons test, selected comparisons were between drug treatments and DMSO control. **D** Representative images of pan-nuclear γH2AX and pKAP1 immunofluorescence staining in OCM.109 after 96 h treatment with 1 μM PARGi. Scale bar: 20 μm. **E** Quantification of RPA foci per nucleus after 48 h of 1 μM PARGi using single-cell immunofluorescence microscopy, left panel normalised to DMSO-treated cells (Control) and represented as fold change. Mean of 3 biological replicates. Error bars represent SEM. Statistics: 2-way ANOVA with Dunnett’s multiple comparisons test, selected comparisons were between drug treatments and DMSO control. **F** Representative images of nuclear RPA foci immunofluorescence staining after 96 h of 1 μM PARGi. Scale bar: 10 μm. **G** Representative immunoblots of pChk1 expression in PARGi-sensitive (109 and 246) and PARGi-resistant (105) OCMs following 96 h of treatment with DMSO (C), 1 μM PARPi (Pi), 1 μM PARGi (Gi) or 10 Gy of ionising radiation (IR). Tao1 serves as loading control. **H** Cell fate profiling, showing cell behaviour over 120 h treatment with 1 μM PARGi or DMSO (Control). See Fig. 3 and Fig. S8. **p < 0.01, ***p < 0.001, ****p < 0.0001

Having identified PARGi-sensitive OCMs, we asked whether they also showed the hallmarks of replication catastrophe upon PARG inhibition. As with the established cell lines, PARGi stabilised PAR chains in both PARGi-sensitive and -resistant OCMs in a PARP-dependent manner (Fig. 6A, B), consistent with the ability of resistant cells to tolerate persistent PAR chains. Importantly, PARGi induced hallmarks of replication catastrophe in two of the three sensitive OCMs analysed (109 and 246), which was evidenced by significant increases in gH2AX, pKAP1, RPA foci, Chk1 phosphorylation and a pre-mitotic cell cycle block (Fig. 6C–H, S8A, B). By contrast, the PARGi-resistant OCMs did not display these hallmarks. Thus, the two OCMs that are sensitive to PARGi, but PARPi-resistant, do indeed undergo replication catastrophe in a manner similar to the PARGi-sensitive established cell lines. Surprisingly, despite OCM.191 being sensitive to PARGi in both short and long-term assays, it did not display the features typical of replication catastrophe. A pre-mitotic block was evident in a small proportion of cells following PARGi treatment and, although most cells underwent mitosis, there were fewer total mitoses in PARGi-treated than control cells (Fig. S8A). This was partly owing to a longer interphase and, despite a similarly high rate of apoptotic events both with and without treatment, apoptosis tended to occur earlier in PARGi-treated cells. In addition, OCM.191 had an intermediate EC_50_, similar to OV56 in the cell line panel, which similarly showed moderate sensitivity to PARGi in short and long-term assays without features of significant replication stress with PARGi-treatment (Fig. 2D, S3).

Interestingly, OCM.191 is also PARPi-sensitive, and indeed it harbours a *BRCA* mutation and is possibly therefore HRD. Because HR defects may contribute to PARGi-sensitivity [22, 24], one possibility therefore is that the defective HR may contribute to PARGi sensitivity in this case. Interestingly, OCM.246 was also derived from a patient with a germline *BRCA2* mutation; however the patient received Olaparib maintenance monotherapy prior to biopsy sampling and the subsequent OCM harbours an intragenic *BRCA2* reversion predicted to restore the open reading frame [51, 52] (Supplementary Table 2), explaining ex vivo PARPi resistance. Consistently, OCM.246 appears to be HRP, evidenced by the accumulation of Rad51 foci in irradiated G2 cells (Fig. S8C). By contrast, in the same assay, OCM.109 appears to be HRD, consistent with the notion that HR status is not a predictor of PARGi sensitivity [26]. Thus, while the relationship between HR status, PARPi and PARGi sensitivity is complex, we nevertheless conclude that the PARGi sensitivity in patient-derived OCMs 109 and 246 is indeed due to the induction of replication catastrophe.

### DNA replication gene expression in OCMs does not correlate with PARGi-sensitivity

**Fig. 7:**
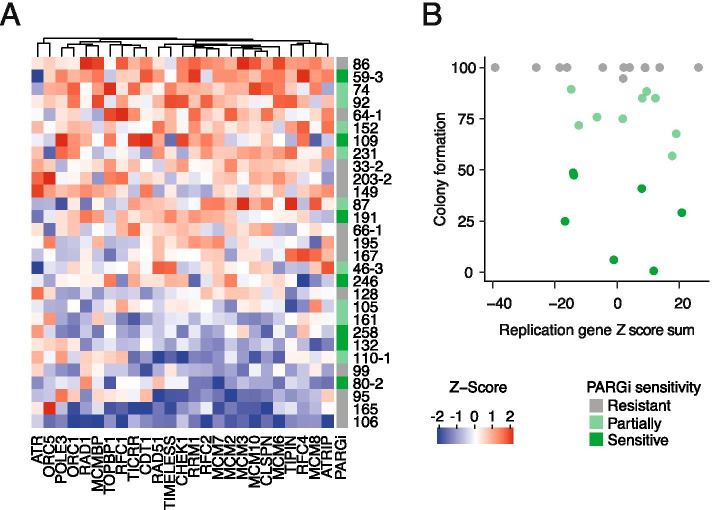
DNA replication gene expression in OCMs does not correlate with PARGi-sensitivity. **A** Heatmap showing RNAseq of 29 OCMs ranked by sum of Z-scores for 25 DNA replication genes where red = high expression; white = average expression and blue = low expression. **B** XY plot showing colony formation area and the sum of Z-scores for the expression of 25 DNA replication genes from RNAseq of 29 OCMs. PARGi sensitivity as determined by colony formation assay (see Fig. S6B). Note: RNAseq was unavailable for OCMs 267, 250–2, and 162–2. See also Fig. S6 and S9

Having demonstrated that PARGi-sensitivity was accompanied by replication catastrophe in two of the three sensitive OCMs examined, we asked whether the expression levels of DNA replication genes correlated with sensitivity, as observed in the established cell lines (Fig. 4B). The DNA replication, mitotic and apoptotic genes examined using NanoString in the established cell lines were evaluated in 29 OCMs for which we have RNAseq data [13, 30, 42]. As with our Nanostring data and the CCLE data, the expression of DNA replication and mitotic genes was highly correlated (Fig. S9A). In addition, there was a gradient in DNA replication expression across the OCM panel, with OCMs 86 and 106 showing the highest and lowest aggregated Z-scores respectively (Fig. 7A). However, ranking the OCMs by overall expression of the DNA replication genes did not differentiate PARGi-sensitive and -resistant OCMs (Fig. 7A, B). Similarly, ranking by mitotic and apoptotic genes also failed to differentiate PARGi sensitivity (Fig. S9B, C). This suggests that, in contrast to our earlier hypothesis [26], a DNA ‘replication stress’ gene expression signature may not be sufficient to serve as a predictive biomarker for PARG inhibitor sensitivity in a clinical cohort of HGSOC. Below we discuss these results in the context of developing a predictive biomarker for PARGi sensitivity.

## Discussion

We set out to address two questions: (1) whether PARGi-sensitive ovarian cancer cells exhibit similar or different phenotypes in response to PARG inhibition, and (2) whether PARGi sensitivity correlates with the expression levels of DNA replication genes. Our analysis of 10 established ovarian cancer cell lines shows that indeed, the four PARGi-sensitive lines all displayed features of persistent replication stress upon exposure to PARGi. Moreover, interrogating three independent gene expression data sets identified a clear correlation between PARGi sensitivity and expression of DNA replication genes. We also observed a replication catastrophe phenotype in two PARGi-sensitive patient-derived ex vivo OCMs; however, in contrast to the established cell lines, they did not display lower DNA replication gene expression relative to PARGi-resistant OCMs. Below, we discuss these observations in terms of (a) the mechanisms responsible for PARGi sensitivity, and (b) efforts to develop biomarkers capable of predicting PARGi sensitivity.

To address the first question, we initially focused on a panel of 10 established ovarian cancer cell lines, four of which are PARGi sensitive and the remaining six, resistant. Upon PARG blockade, the four sensitive lines displayed features of replication stress and DNA damage, namely RPA and γH2AX foci, phosphorylation of Kap1 and Chk1, and asymmetric DNA replication forks, in turn leading to replication catastrophe illustrated by pan-nuclear γH2AX, pre-mitotic cell cycle arrest, reduced proliferation and suppressed outgrowth. By contrast, the resistant lines did not display these hallmarks, despite the stabilisation of PAR chains. Of the three PARGi-sensitive OCMs we analysed in detail, two also exhibited hallmarks of replication catastrophe, extending our observations from established cell lines to patient-derived ovarian cancer cells that have not undergone extensive ex vivo proliferation [13]. Together, this recurrent replication catastrophe phenotype supports our prior hypothesis that PARGi-sensitivity arises due to a pre-existing DNA replication vulnerability that prevents toleration of stabilised PAR chains. The nature of this vulnerability remains unclear but could reflect compromised replisome function, a notion supported by reduced DNA replication gene transcripts in sensitive lines. However, whether these genes are *down-regulated* due to active suppression of transcription is not clear. Alternatively, resistant cell lines may have actively *up-regulated* DNA replication genes, possibly as an adaptive response to oncogene-induced replication stress [53, 54]. In budding yeast, various adaptive mechanisms can increase fitness in response to replication stress [55], raising the possibility that differential PARGi sensitivity reflects different responses to oncogenic replication stress. Thus, while adaption via up-regulating DNA replication genes could engender intrinsic PARGi resistance, adaptation via other mechanisms may leave cells vulnerable to PARG blockade. Exploring the evolution of acquired PARGi resistance may shed light on this issue.

While our data strongly points to a DNA replication vulnerability, other mechanisms can also render cells dependent on PARG activity. By sequestering NAD+ in stabilised PAR chains, PARG inhibition was recently shown to cause metabolic catastrophe in glioma cell lines harbouring *IDH1* mutations [56]. This study employed the same PARG inhibitor but with sensitivity observed at a five-fold higher drug concentration and only when used in combination with temozolomide, indicating that in this case, sensitivity is due to a ‘*triple whammy*’ effect requiring *IDH1* mutation, DNA damage and PAR stabilisation. Interestingly, in our study, partial PARGi sensitivity in OV56 was not accompanied by overt replication stress. Similarly, PARGi-sensitive OCM.191 did not show signs of replication stress with treatment. Whether this intermediate sensitivity is accounted for by NAD+ sequestration and metabolic catastrophe remains to be seen. Moreover, whether PARGi-induced metabolic catastrophe is distinct from or related to the DNA replication vulnerability mechanism is also unclear. Indeed – because, firstly NAD+ metabolism is required to produce NADP+, and subsequently nucleic acid precursors via the pentose-phosphate pathway [57, 58], and secondly nucleotide depletion increases replication stress – PARGi sensitivity could reflect a complex interplay between DNA replication and NAD+ vulnerabilities. For example, at low doses, PARGi may deplete NAD+ sufficiently to perturb nucleotide synthesis resulting in replication stress that can only be buffered by cells that have adapted to oncogene-induced replication stress. By contrast, when combined with acute DNA damage at higher doses, PARGi may deplete NAD+ to the point that it perturbs NAD+ pathways more broadly, leading to metabolic catastrophe. An interesting corollary therefore is that PARGi-sensitising DNA replication vulnerabilities could arise due to upstream defects in supplying nucleotides to the replisome, which are exacerbated by PARG blockade. Exploring the interplay between NAD+ metabolism, nucleotide synthesis and DNA replication in the context of PAR dynamics will be important next steps.

Our second question regarding a potential correlation between PARGi sensitivity and expression levels of DNA replication genes was motivated by the quest to develop predictive biomarkers for PARGi sensitivity to enable patient stratification for clinical trials. Interrogating 25 DNA replication genes in two independent RNAseq datasets and our NanoString analysis shows a clear correlation; the four PARGi-sensitive ovarian cancer cell lines show reduced expression levels versus the resistant lines. If this correlation extended to patient-derived OCMs, then one could envision a ‘*replication stress*’ gene expression signature providing the basis of a biomarker [26]. However, we did not observe a similar effect in PARGi-sensitive OCMs. There are various possible explanations for the non-concordance between established cell lines and OCMs. One possibility is sample size; while the colony formation screen identified seven OCMs as potentially sensitive, only OCMs 109 and 246 were particularly sensitive with nanomolar EC_50_ values. As our living biobank grows, it will be important therefore to identify additional PARGi-sensitive OCMs to further test whether DNA replication gene expression correlates with sensitivity. A second possibility is that analysing established cell lines is complicated by different ovarian cancer subtypes. While PARGi-sensitive Kuramochi is HGSOC-derived, the three other sensitive cell lines (OVMANA, OVISE and RMG1) represent the clear cell subtype [30, 45]. Thus, the correlation may reflect a clear cell phenomenon not shared more broadly with HGSOC. Interestingly, OCMs 80–2 and 87 may be clear-cell-derived [30], and while OCM.80–2 is PARGi sensitive and has reduced DNA replication transcripts, OCM.87 is more resistant and does not, reinforcing the need to analyse PARGi sensitivity in a broad range of OCMs.

Another complication extending observations from established cell lines to OCMs is chromosome instability (CIN). Although ovarian cancer cell lines exhibit CIN, it is markedly reduced compared with the mitotic chaos observed in primary cultures, most probably because extended ex vivo propagation selects fitter, relatively stable clones [13, 59]. Constant chromosome reshuffling likely generates transcript heterogeneity, in turn influencing drug sensitivity and the stability of gene expression signatures [60–62]. Interestingly, the two PARGi-sensitive OCMs that displayed PARGi-induced replication stress (109 and 246) have karyotypes dominated by relatively stable monosomies and disomies, compared with the highly variable chromosome gains and focal amplifications typical of resistant OCMs (Fig. S5F). Exploring the relationship between karyotypic features, CIN and PARGi sensitivity will also be facilitated by a broad range of well-characterised OCMs.

And finally, the correlation between replisome gene expression and PARGi sensitivity is partly a circular argument. Our initial focus on replisome genes was motivated by (a) the replication catastrophe phenotype, and (b) the emergence of replisome genes in a PARGi-sensitiser RNAi screen [26]. In turn, interrogating replisome genes led us to test sensitivity of OVMANA, OVISE and RMG1, which we now confirm have relatively low expression levels of DNA replication genes. This highlights the need to take fresh, unbiased approaches on larger collections of OCMs with defined drug sensitivity. Indeed, it is striking that the expression of S-phase and mitotic genes correlates strongly, not only in the panel of 10 established cell lines but generally across the CCLE dataset and our cohort of OCMs. This raises the possibility that the differential gene expression between PARGi-sensitive and -resistant cell lines is mediated by a common upstream regulatory network, e.g. the E2F network, which drives both S-phase and mitotic gene expression programs [63]. Indeed, E2F network genes, along with DNA replication and mitotic spindle genes, are up-regulated following introduction of *TP53* and *BRCA1* mutations in a cell line model of ovarian cancer development, corresponding with the emergence of CIN [64]. Alternatively, correlated expression of S-phase and mitotic genes may independently arise in response to the same selective pressure, namely oncogene-induced replication stress. The stronger correlation observed for our cell line panel, than in cancer cell lines generally, in a disease characterised by high levels of CNV, implies that a common evolutionary pressure is a more likely explanation. Interestingly, in the yeast model alluded to above, in addition to DNA replication processes, sister chromatid cohesion networks were also repeatedly altered by exposure to replication stress [55]. PARGi-sensitivity may therefore reflect a broader adaptation of cell cycle processes that arise in response to oncogene-induced deregulation of cell cycle controls.

Synthesising these issues, we conclude that the relationship between the expression levels of replisome genes and PARGi sensitivity is more complex than initially proposed [26]. Therefore, developing robust predictive biomarkers with potential for clinical utility will require more mechanistic insight, driven by both hypothesis-led and unbiased approaches. Nevertheless, a key outcome of this study is the identification of two OCMs that are particularly sensitive to single-agent PARG inhibition. While we previously described OCMs sensitive to PARGi in combination with a CHK1 inhibitor [26], these latter observations indicate that PARG inhibitors may have efficacy as monotherapies. This is encouraging because there is a need for new therapeutic agents, with appropriate predictive biomarkers, and in particular for the large cohort of women with HRP HGSOC who are unlikely to benefit from PARP inhibitors. A key question therefore is whether PARG inhibitors will offer distinct therapeutic opportunities to PARP inhibitors. Interestingly, our screen identified a similar proportion of OCMs with PARGi and PARPi sensitivity, and of seven PARGi-sensitive OCMs, four were PARPi-resistant, consistent with previous reports that PARGi and PARPi sensitivity are mostly non-overlapping [26, 28]. Indeed, of the six PARGi-resistant established cell lines used in this study, four are sensitive to Olaparib, and of four PARGi-sensitive cell lines, only two are sensitive to Olaparib. Non-overlapping sensitivity to PARPi and PARGi may seem counter-intuitive, as PARP1/2 and PARG work in concert to repair DNA damage; one might expect that both PARPi and PARGi would be toxic towards tumour cells with defects in DNA damage repair [22]. However, while some studies suggest synthetic lethality between *BRCA1/2* mutations and PARG inhibition [22, 24] others do not [26, 65]. Indeed, two of the PARGi-resistant cell lines, COV362 and OVSAHO, have reported *BRCA* defects [45, 66, 67] and are PARPi-sensitive. Moreover, in terms of PARGi-sensitive lines, OVMANA, Kuramochi, RMG1 and OCM.109 have *BRCA1/2* mutations or deletions, while OVISE is *BRCA1/2* wildtype and OCM.246 has a *BRCA2* reversion. Thus, our analysis confirms that *BRCA* and HR status do not predict PARGi sensitivity, highlighting the need for more mechanistic insight to drive biomarker development.

## Conclusions

Here we show that in a panel of established cell lines, PARGi sensitivity is accompanied by hallmarks of replication catastrophe, and correlates with lower expression of DNA replication genes. We also identify several patient-derived OCMs that are sensitive to PARGi monotherapy, again via a DNA replication catastrophe mechanism. However, DNA replication gene expression did not correlate with sensitivity of the OCMs meaning a DNA ‘replication stress’ gene expression signature is unlikely to be a sufficient predictive biomarker for PARG inhibitor sensitivity in a clinical cohort of HGSOC. These results highlight the complexity of developing a predictive biomarker for PARGi sensitivity. Thus, while further research is required to delineate mechanisms of PARGi sensitivity and to develop predictive biomarkers, this study nonetheless reinforces the potential of PARG as a new therapeutic target for women with HGSOC, including those who develop platinum-resistant disease who currently have an overall survival of only ~ 12 months [68]. Significantly, OCMs 109 and 246, the two most highly PARGi-sensitive, were biopsied from women with platinum-resistant disease. Moreover, although patient 246 was previously treated with Olaparib maintenance monotherapy, OCM.246 has a *BRCA2*-reversion, is HRP and PARPi-resistant. Thus, this study indicates that PARG inhibitors may represent a future alternative treatment for patients with otherwise limited therapeutic options, such as those with disease that is platinum and/or PARPi resistant, and informs the design of early clinical studies of PARG inhibitors.

## Supplementary Information

**Additional file 1: Fig. S1.** Ovarian cancer cell lines exhibit differential sensitivity to PARGi and PARPi. (A) Colony formation in the continuous presence of 1 μM PARGi, 1 μM PARPi or DMSO (Control). Representative of ≥3 biological replicates. (B) Quantification of colony area with constant PARGi treatment or (C) 24–72 h wash-out PARGi treatment, normalised to DMSO-treated cells (Control) and represented as fold-change. Mean of ≥3 biological replicates. Samples below dotted line have > 80% reduction in colony formation. (D) Exemplar PARGi-resistant (OVSAHO) and PARGi-sensitive (OVMANA) cell proliferation curves (measured as green object count, GOC), at increasing concentrations of PARGi. Mean of 2 biological replicates. (E) AUC from (D) were used to dose-response curves shown. Mean of 2 biological replicates. (F) Proliferative EC_50_ values for the cell line panel. Mean of ≥2 biological replicates. PRISM could not accurately calculate EC_50_ for resistant cells, therefore for highly resistant cell lines EC_50_ was approximated as 50 μM (half the maximal concentration tested), and for less highly resistant OV56, EC_50_ was determined manually. (G) Quantification of colony area in response to continuous PARPi treatment quantified normalised to DMSO-treated cells (Control) and represented as fold-change. Samples below dotted line have > 80% reduction in colony formation. Mean of ≥3 biological replicates. Statistics: 2-way ANOVA with Dunnett’s multiple comparisons test, selected comparisons were between drug treatments and DMSO control within each cell line. Error bars represent SEM. (H) Venn diagram summarising differential sensitivity. *p < 0.05, **p < 0.01, ***p < 0.001, ****p < 0.0001.

**Additional file 2: Fig. S2.** PARGi stabilises PAR chains in cell lines irrespective of PARGi sensitivity. (A) Quantification of PAR staining intensity in response to 48 h treatment with DMSO (Control), 1 μM PARGi, and co-treatment with 1 μM PARGi and 1 μM PARPi using single-cell immunofluorescence microscopy in 1 biological replicate (dot plots, 1000 cells shown per condition). (B) Representative immunofluorescence images of PAR staining from (A), in PARGi-resistant (COV318) and PARGi-sensitive (OVMANA) cell lines. Scale bar: 20 μm. (C) Quantification of PAR staining in response to PARGi or co-treatment with PARGi and PARPi, normalised to DMSO-treated cells (Control). Mean of ≥3 biological replicates. (D) Representative immunoblot showing PAR chain formation in response to 48 h treatment with DMSO (Control), or 1 μM PARGi. (E) PAR immunoblot in (D), without adjustment to show inter-line variation. (F) Mean quantification of PAR staining by immunoblotting (≥2 biological replicates). Statistics: 2-way ANOVA with Dunnett’s multiple comparisons test, selected comparisons were between drug treatments and DMSO control. Error bars represent SEM. * *p* < 0.05, ** *p* < 0.01, *** *p* < 0.001, **** *p* < 0.0001.

**Additional file 3: Fig. S3.** PARGi sensitivity is accompanied by markers of replication stress and the DNA damage response. (A) Quantification of foci per nucleus after 48 h of 1 μM PARGi treatment (γH2AX and RPA1), or nuclear pKAP1 intensity after 72 h of 1 μM PARGi treatment using single-cell immunofluorescence microscopy in 1 biological replicate (dot plots, 1000 cells shown per condition). (B) Upper panel: Quantification of foci per nucleus after 48 h of 1 μM PARGi treatment (γH2AX and RPA1), or nuclear pKAP1 intensity after 72 h of 1 μM PARGi treatment; Lower panel: Results from upper panel normalised to DMSO-treated cells (Control) and represented as fold-change. Mean of ≥3 biological replicates. (C) Representative immunoblot for Chk1 and pChk1, following 48 h with 1 μM PARGi (Gi) or DMSO as a negative control (C), or for 2 h with 2 mM hydroxyurea (H) as a positive control. Tao1 serves as loading control. (D) Quantification of Li-COR pChk1 immunoblotting shown in Fig. 2C, mean of ≥3 biological replicates. Data are expressed as the increase resulting from treatment as a percentage of maximum response (achieved with hydroxyurea [H]) to correct for inter-line variation i.e. % PARGi (pChk1/Chk1)/H - % DMSO (pChk1/Chk1)/H. Statistics: 2-way ANOVA with Sidak multiple comparisons test (B, D), selected comparisons were between PARGi treated values and DMSO control within each cell line. Error bars represent SD (A), SEM (B, D). *p < 0.05, **p < 0.01, ***p < 0.001, ****p < 0.0001.

**Additional file 4: Fig. S4.** Comparison of NanoString with CCLE and Klijn datasets; DNA replication and mitotic gene expression correlation. (A) Box and whisker plots for DNA replication (red), mitotic (blue) and apoptotic (grey) gene Z scores in 10 cell line panel. Values on left side indicate Pearson R^2^ for CCLE or data from Klijn et al., compared with the NanoString analysis. (B) XY plot of the correlation between DNA replication and mitotic (red), and DNA replication and apoptotic (grey) gene expression. Z scores sums for epithelial cancer cell lines in Broad 2019 CCLE dataset [39]. Ten cell line panel indicated by black diamonds, note OV56 CCLE expression data does not reflect NanoString expression data (see text). Pearson R^2^ values indicated below the graph, all comparisons, p < 0.0001, total number cell lines 747. ns = not significant, *p < 0.05, **p < 0.01, ***p < 0.001, ****p < 0.0001.

**Additional file 5: Fig. S5.** Validation of patient-derived OCMs as bona fide models of HGSOC. (A) Representative images of severely atypical nuclei seen across OCMs. Scale bar: 10 μm. (B) Representative images of CK7 and PAX8, and p53 mutation-type (OCM.106: absent nuclear expression; OCM.195: strong/diffuse nuclear expression, involving > 80% tumour cell nuclei) by immunofluorescence staining. Scale bar: 20 μm. (C) Representative images of CK7, PAX and p53 mutation-type (OCM.92: absent nuclear expression; OCM.191: strong/diffuse nuclear expression, involving > 80% tumour cell nuclei) by immunohistochemistry staining from archival tumour blocks. Scale bar: 500 μm (× 10 magnification) and 100 μm (× 40 magnification). (D) Representative p53 immunoblot e.g. showing absent (OCM.86) and strong p53 bands (OCM.105). The control well represents stromal cells from the patient sample associated with OCM.237. Tao1 serves as loading control. (E) Representative somatic *TP53* variants detected in OCMs. (F) Exemplar images of genome-wide chromosome copy-number profiles determined by single-cell whole-genome sequencing showing aneuploidies and rearranged chromosomes in tumour cells. Each row represents a single cell, with chromosomes plotted as columns and colours depicting copy-number state. See also Supplementary Table 1.

**Additional file 6: Fig. S6.** Living biobank screen demonstrated broad range of PARGi and PARPi sensitivity. (A) Colony formation following 96 h of treatment with 1 μM PARGi or 1 μM PARPi or DMSO (Control). (B) Quantification of ca from (A) following 1 μM PARGi (B) or 1 μM PARPi (C), normalised to DMSO-treated cells (Control) and represented as fold-change. BRCA status (germline, or, where available, OCM) indicated as follows: + = BRCA status of OCM; W=*BRCA1/2* wild-type; *B*=*BRCA1/2* mutation; V=*BRCA1/2* variant of uncertain clinical significance; R = putative *BRCA1/2* reversion; ▼=prior PARPi therapy. Single technical replicate. (D) Exemplar images of colony formation following continuous treatment with 1 μM PARGi or 1 μM PARPi or DMSO (Control). Representative images of 3 biological replicates. (E) Quantification of ca from (D) normalised to DMSO-treated cells (Control) and represented as fold-change. Mean of 3 biological replicates. Error bars represent SEM. See also Supplementary Table 2 and Fig. 5A, B. ***p < 0.001, ****p < 0.0001.

**Additional file 7: Fig. S7.** On-target inhibition of PARG in PARGi-sensitive OCMs. (A) Chemical structure of inactive small molecule analog of PARGi, PARGi-Me (PDD00031704). (B) Colony formation of PARGi-sensitive OCMs (109, 191 and 246) following continuous treatment with 1 μM PARGi or 1 μM PARGi-Me or DMSO (Control). Representative images of 3 biological replicates. (C) Upper panel – Proliferative Log EC_50_ values for PARGi and PARGi-ME for PARGi-sensitive OCMs. Mean of 3 biological replicates. Error bars represent SEM. Statistics: Unpaired t-test of PARGi versus PARGi-Me. Lower panel – Quantification of ca from (B) normalised to DMSO-treated cells (Control) and represented as fold-change. Mean of 3 biological replicates. Statistics: Unpaired t-test of PARGi versus PARGi-Me. Error bars represent SEM. ***p < 0.001, ****p < 0.0001.

**Additional file 8: Fig. S8.** PARGi suppresses mitotic entry in sensitive OCMs. (A) Cell fate profiling, showing cell behaviour over 120 h treatment with 1 μM PARGi or DMSO (Control). Each horizontal line represents a single cell, with the colours indicating cell behaviour. Following mitosis, one daughter cell was chosen at random to continue the analysis. No mitotic entry does not include death in interphase where mitosis does not take place. Abnormal mitosis includes cell division, abnormalities such as tripolar cell divisions, binuclear daughter cells and division of binuclear cells. Slippage is recorded where cells enter mitosis, then exit without division. Fusion was recorded where daughter cells appear to separate but subsequently join back together. Representative of 2 biological replicates. (B) Number of cells that fail to enter mitosis over 120 h (maximum *n* = 50) following 1 μM PARGi, 1 μM PARPi or DMSO (Control). Statistics: 2-way ANOVA with Dunnett’s multiple comparisons test, selected comparisons were between PARGi or PARPi treated cells versus DMSO control. Mean of 2 biological replicates. Error bars represent SEM. (C) Exemplar image of CENPF (indicating G_2_ cells) and Rad51 immunofluorescence staining of OCM.246 after 2 Gy X-ray ionising radiation followed by 24 h PARPi, ▼ indicates an HRP cell (positive for CENPF with > 5 Rad51 foci). Scale bar: 10 μm. Bar chart shows fold-change in % [Rad51 + CENPF+ cells/CENPF+ cells] in DMSO-treated cells versus cells treated with 2 Gy X-ray ionising radiation followed by 24 h 1 μM PARPi. Dotted line indicates a 2-fold change, above which cells are considered HRP (below it HRD).

**Additional file 9 Fig. S9.** Low expression of mitotic or apoptotic gene sets does not identify OCMs sensitive to PARGi. (A) XY plot showing correlation between sum of Z scores for mitotic and DNA replication genes for each OCM (p < 0.0001, Pearson’s R^2^ = 0.774). (B) Heatmaps showing RNAseq of 29 OCMs ranked by sum of z-scores for 25 mitotic (left) and apoptotic (right) genes where red = high expression; white = average expression; and blue = low expression. (C) XY plots showing colony formation area and the sum of z-scores for the expression of 25 mitotic (left) and apoptotic (right) genes from RNAseq of 29 OCMs. PARGi sensitivity as determined by colony formation assay (see Fig. S6B). Note: RNAseq was unavailable for OCMs 267, 250–2, and 162–2.

**Additional file 10: Supplementary Table 1.** Clinical data, OCMs and primary tumour blocks. The table outlines the clinical data for the 25 new HGSOC OCMs screened for PARPi and PARGi sensitivity. Key: d_x_, diagnosis; FIGO, International Federation of Gynecology and Obstetrics; g*BRCA*m, germline *BRCA1/2* mutation; VUS, variant of uncertain clinical significance; CTx, chemotherapy; Ref, platinum-refractory disease (tumour progression during or within 4 weeks of completing platinum therapy); Res, platinum-resistant (tumour progression between 4 weeks and 6 months from completing platinum therapy); Sens, platinum-sensitive (tumour progression ≥6 months from completing platinum therapy). CN, chemonaïve; OS, overall survival; mo, months; WT, wild-type; FR, frameshift; NS, nonsense; IF, immunofluorescence; IHC, immunohistochemistry; For IF and IHC: CK7 and PAX8, coloured box (present), white box (absent), S, strong; P, patchy; F, focal; W, weak. p53 is either mutant-type (strong/diffuse nuclear staining; darker coloured box), wild-type (lighter colour box) or absent nuclear staining (white box). NE, not evaluable (antibody failed); block unavailable (grey box). IB, immunoblotting; For IB: +(sm), band present but at lower than 53 kDa; +(S), strong band present; *At the time of the research biopsy; ^†^Histologically re-classified from HGSOC to intermediate grade (grade 2/moderately differentiated) serous adenocarcinoma following tumour block analysis; ^‡^cell agar block only, histologically re-classified from HGSOC to suspicion of adenocarcinoma arising from the gynaecological tract. Clinical data for previously characterised OCMs are published [13], and not repeated here. **Supplementary Table 2.** *BRCA1/2* variants detected in OCMs. The table outlines the *BRCA1/2* variants detected in the panel of 7 OCMs screened for PARGi and PARPi sensitivity using colony survival and cell proliferation assays. Variants are described using Human Genome Variation Society (HGVS) nomenclature. Key: FR, frameshift; MS, missense; NS, nonsense; VAF, variant allele frequency; VUS, variant of uncertain clinical significance; WT, wild-type. All OCMs underwent both NGS and MLPA testing for *BRCA1/2* variants. **Supplementary Table 3**. EC_50_ values for OCMs for the inhibitors/compounds tested. The table outlines the EC_50_ values for the inhibitors/compounds tested in the cell proliferation assay for the panel of 7 OCMs assessed. See Fig. 5C and D for Log EC_50_ values. Data are mean ± SEM from 3 biological replicates. PRISM could not accurately calculate EC_50_ for PARGi-resistant cells, therefore for resistant OCMs, the EC_50_ was approximated as 50 μM (half the maximal concentration tested) and described in the table as not calculable (NC).

## Data Availability

Additional RNAseq datasets generated here from 8 of the novel OCMs are available from EBML-EBI using accession number E-MTAB-11000 and the FASTQ files are available from the European Nucleotide Archive (https://www.ebi.ac.uk/ena/browser/view/E-MTAB-11000) [42]. scWGS karyotyping data generated here from 6 OCMs are available from EMBL-EBI using accession number PRJEB47696 and the FASTQ files are available from the European Nucleotide Archive (https://www.ebi.ac.uk/ena/browser/view/PRJEB47696) [69]. Additional datasets used in this study have been published previously and are available from EMBL-EBI, including RNAseq from 21 additional OCMs (E-MTAB-7223 and E-MTAB-10801) and scWGS karyotyping data for OCM.46–3 (PRJEB28664) [13, 30].

## Abbreviations

ca: colony area
CCLE: Cancer Cell Line Encyclopedia
CIN: chromosome instability
CNV: copy number variation
EC_50_: half maximal effective concentration
FFPE: formalin-fixed and paraffin-embedded
HGSOC: high-grade serous ovarian cancer
HR: homologous recombination
HRD: homologous recombination deficient
HRP: homologous recombination proficient
HU: hydroxyurea
MCRC: Manchester Cancer Research Centre
MLPA: multiplex ligation probe amplification
NGS: next-generation sequencing
OCM: ovarian cancer model
PAR: poly(ADP-ribose)
PARG: poly(ADP-ribose) glycohydrolase
PARGi: PARG inhibitor
PARP: poly(ADP-ribose) polymerase
PARPi: PARP inhibitor
RNAseq: RNA sequencing
