# Supplementary figures and images for: Replication catastrophe is responsible for intrinsic PAR glycohydrolase inhibitor-sensitivity in patient-derived ovarian cancer models
Source: J Exp Clin Cancer Res. 2021 Oct 16;40:323. doi: 10.1186/s13046-021-02124-0 (PMC8520217; doi:10.1186/s13046-021-02124-0)

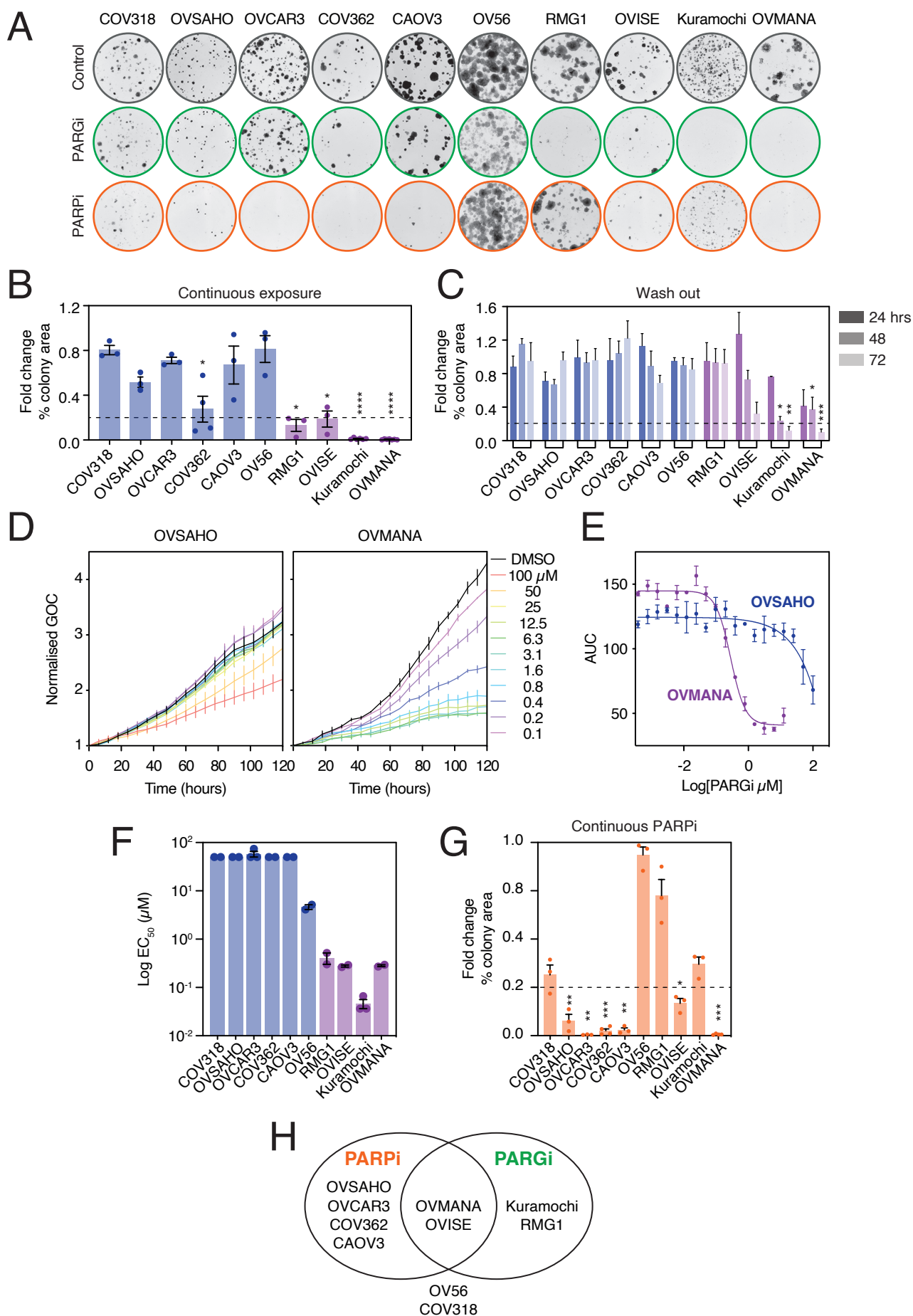

Figure S1

Supplement: Supplementary file 1 — Additional file 1: Fig. S1. Ovarian cancer cell lines exhibit differential sensitivity to PARGi and PARPi. (A) Colony formation in the continuous presence of 1 μM PARGi, 1 μM PARPi or DMSO (Control). Representative of ≥3 biological replicates. (B) Quantification of colony area with constant PARGi treatment or (C) 24–72 h wash-out PARGi treatment, normalised to DMSO-treated cells (Control) and represented as fold-change. Mean of ≥3 biological replicates. Samples below dotted line have > 80% reduction in colony formation. (D) Exemplar PARGi-resistant (OVSAHO) and PARGi-sensitive (OVMANA) cell proliferation curves (measured as green object count, GOC), at increasing concentrations of PARGi. Mean of 2 biological replicates. (E) AUC from (D) were used to dose-response curves shown. Mean of 2 biological replicates. (F) Proliferative EC50 values for the cell line panel. Mean of ≥2 biological replicates. PRISM could not accurately calculate EC50 for resistant cells, therefore for highly resistant cell lines EC50 was approximated as 50 μM (half the maximal concentration tested), and for less highly resistant OV56, EC50 was determined manually. (G) Quantification of colony area in response to continuous PARPi treatment quantified normalised to DMSO-treated cells (Control) and represented as fold-change. Samples below dotted line have > 80% reduction in colony formation. Mean of ≥3 biological replicates. Statistics: 2-way ANOVA with Dunnett’s multiple comparisons test, selected comparisons were between drug treatments and DMSO control within each cell line. Error bars represent SEM. (H) Venn diagram summarising differential sensitivity. *p < 0.05, **p < 0.01, ***p < 0.001, ****p < 0.0001. [file 13046_2021_2124_MOESM1_ESM.pdf]

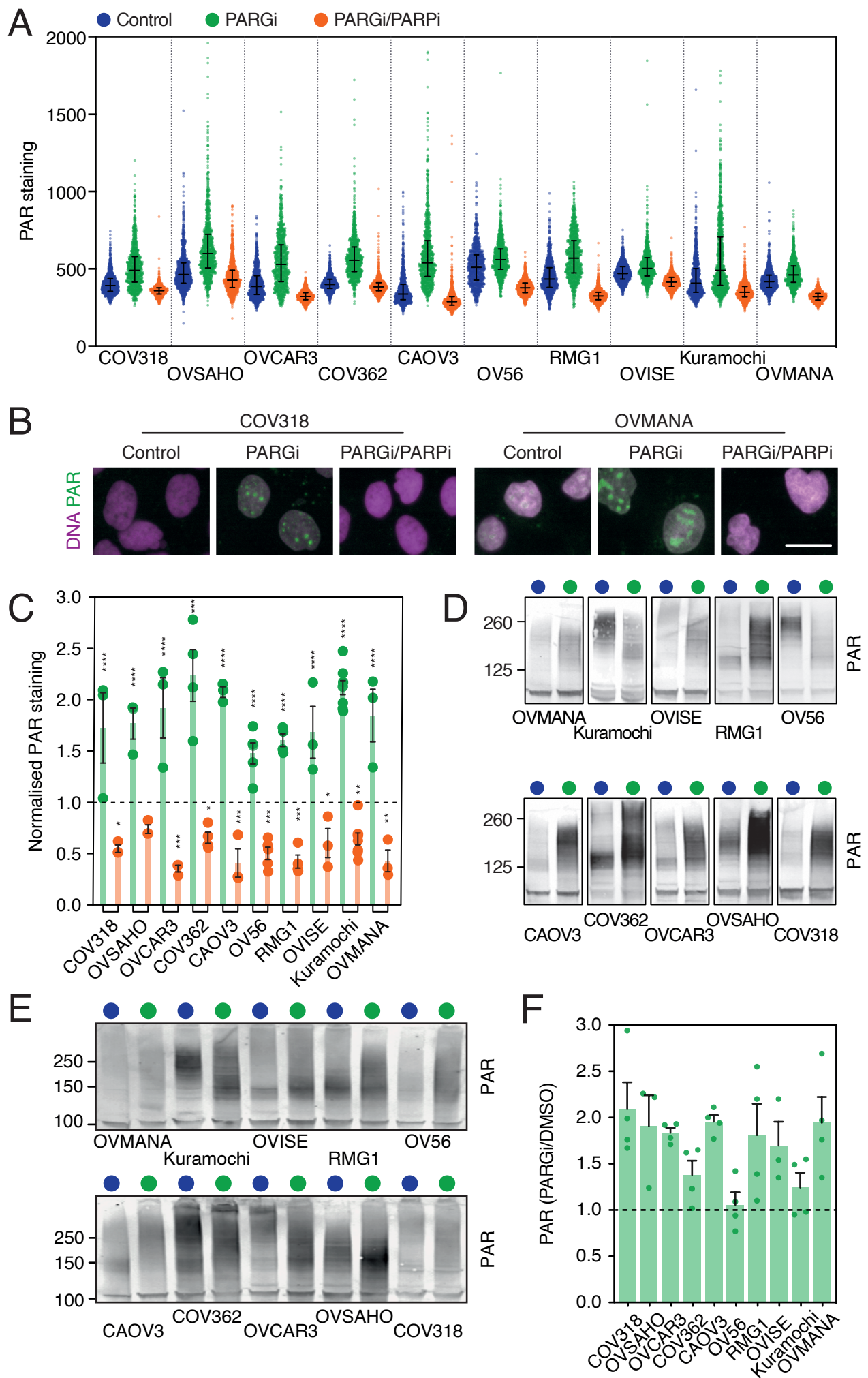

Figure S2

Supplement: Supplementary file 2 — Additional file 2: Fig. S2. PARGi stabilises PAR chains in cell lines irrespective of PARGi sensitivity. (A) Quantification of PAR staining intensity in response to 48 h treatment with DMSO (Control), 1 μM PARGi, and co-treatment with 1 μM PARGi and 1 μM PARPi using single-cell immunofluorescence microscopy in 1 biological replicate (dot plots, 1000 cells shown per condition). (B) Representative immunofluorescence images of PAR staining from (A), in PARGi-resistant (COV318) and PARGi-sensitive (OVMANA) cell lines. Scale bar: 20 μm. (C) Quantification of PAR staining in response to PARGi or co-treatment with PARGi and PARPi, normalised to DMSO-treated cells (Control). Mean of ≥3 biological replicates. (D) Representative immunoblot showing PAR chain formation in response to 48 h treatment with DMSO (Control), or 1 μM PARGi. (E) PAR immunoblot in (D), without adjustment to show inter-line variation. (F) Mean quantification of PAR staining by immunoblotting (≥2 biological replicates). Statistics: 2-way ANOVA with Dunnett’s multiple comparisons test, selected comparisons were between drug treatments and DMSO control. Error bars represent SEM. * p < 0.05, ** p < 0.01, *** p < 0.001, **** p < 0.0001. [file 13046_2021_2124_MOESM2_ESM.pdf]

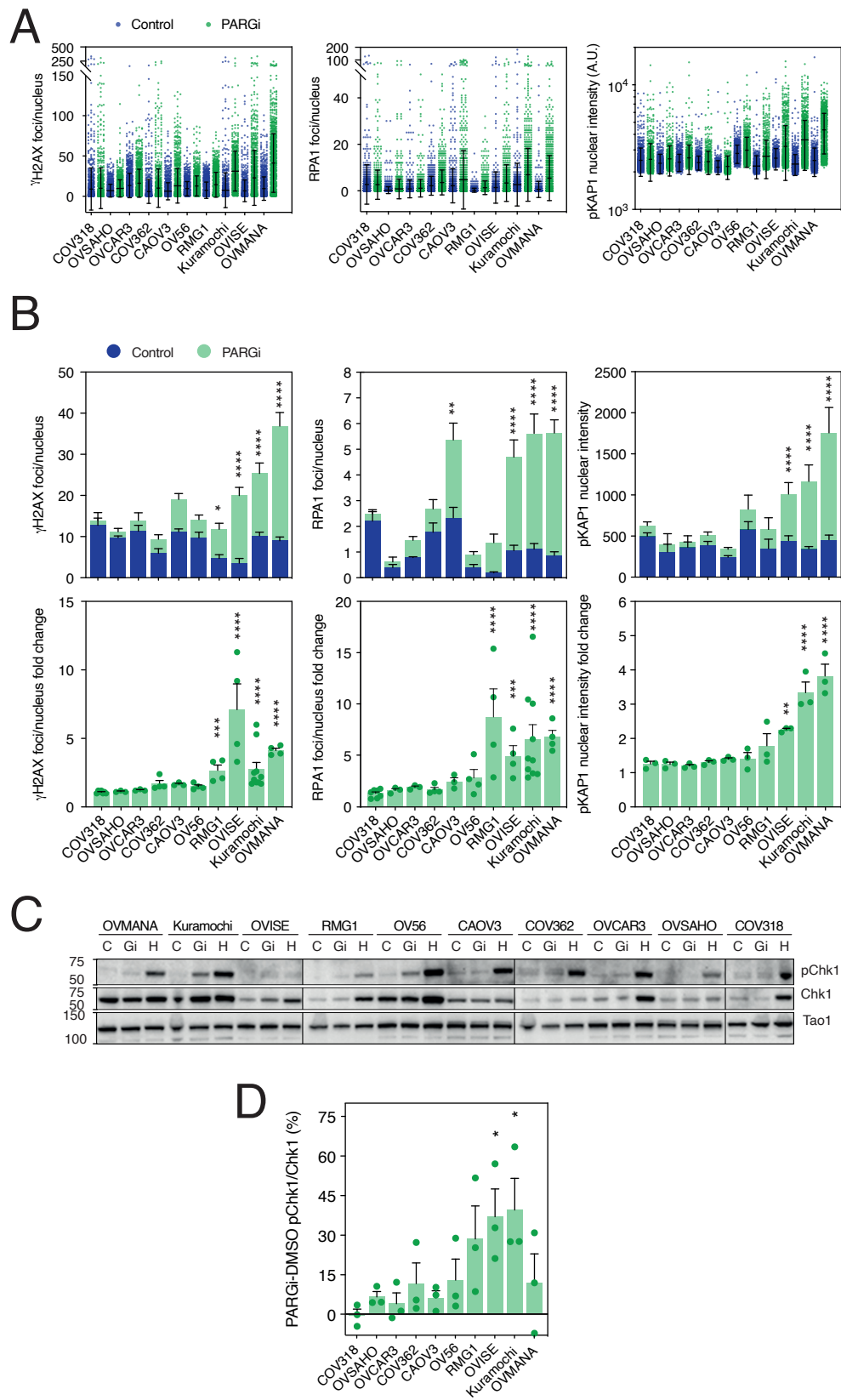

Figure S3

Supplement: Supplementary file 3 — Additional file 3: Fig. S3. PARGi sensitivity is accompanied by markers of replication stress and the DNA damage response. (A) Quantification of foci per nucleus after 48 h of 1 μM PARGi treatment (γH2AX and RPA1), or nuclear pKAP1 intensity after 72 h of 1 μM PARGi treatment using single-cell immunofluorescence microscopy in 1 biological replicate (dot plots, 1000 cells shown per condition). (B) Upper panel: Quantification of foci per nucleus after 48 h of 1 μM PARGi treatment (γH2AX and RPA1), or nuclear pKAP1 intensity after 72 h of 1 μM PARGi treatment; Lower panel: Results from upper panel normalised to DMSO-treated cells (Control) and represented as fold-change. Mean of ≥3 biological replicates. (C) Representative immunoblot for Chk1 and pChk1, following 48 h with 1 μM PARGi (Gi) or DMSO as a negative control (C), or for 2 h with 2 mM hydroxyurea (H) as a positive control. Tao1 serves as loading control. (D) Quantification of Li-COR pChk1 immunoblotting shown in Fig. 2C, mean of ≥3 biological replicates. Data are expressed as the increase resulting from treatment as a percentage of maximum response (achieved with hydroxyurea [H]) to correct for inter-line variation i.e. % PARGi (pChk1/Chk1)/H - % DMSO (pChk1/Chk1)/H. Statistics: 2-way ANOVA with Sidak multiple comparisons test (B, D), selected comparisons were between PARGi treated values and DMSO control within each cell line. Error bars represent SD (A), SEM (B, D). *p < 0.05, **p < 0.01, ***p < 0.001, ****p < 0.0001. [file 13046_2021_2124_MOESM3_ESM.pdf]

A

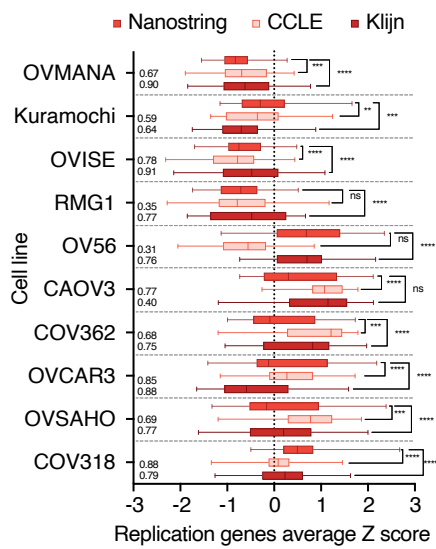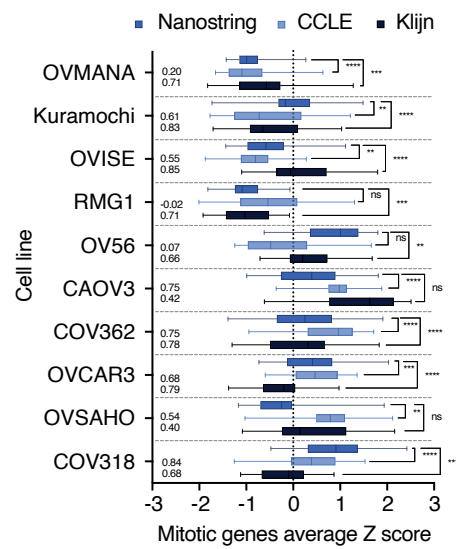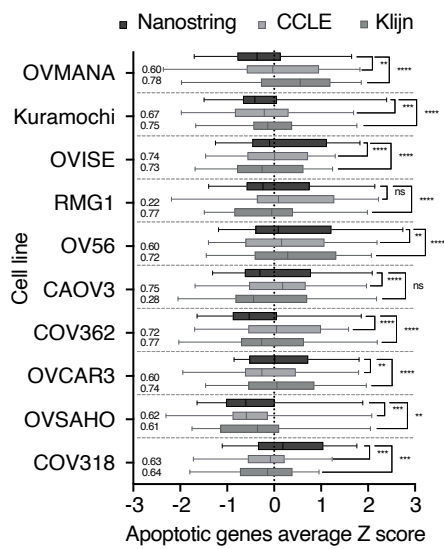

B

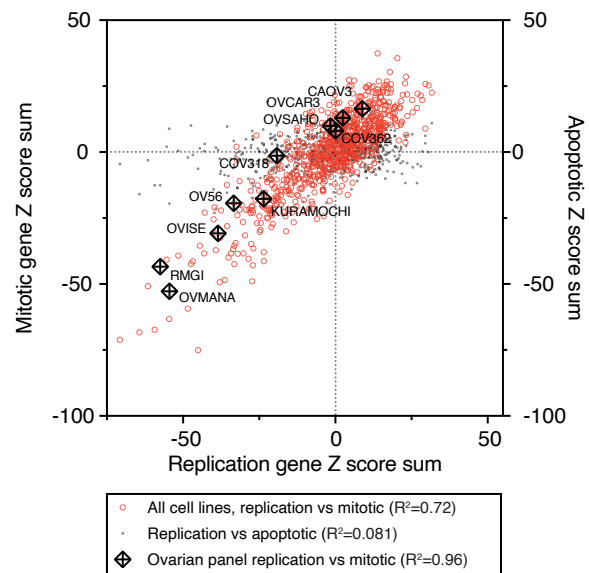

Figure S4

Supplement: Supplementary file 4 — Additional file 4: Fig. S4. Comparison of NanoString with CCLE and Klijn datasets; DNA replication and mitotic gene expression correlation. (A) Box and whisker plots for DNA replication (red), mitotic (blue) and apoptotic (grey) gene Z scores in 10 cell line panel. Values on left side indicate Pearson R2 for CCLE or data from Klijn et al., compared with the NanoString analysis. (B) XY plot of the correlation between DNA replication and mitotic (red), and DNA replication and apoptotic (grey) gene expression. Z scores sums for epithelial cancer cell lines in Broad 2019 CCLE dataset [39]. Ten cell line panel indicated by black diamonds, note OV56 CCLE expression data does not reflect NanoString expression data (see text). Pearson R2 values indicated below the graph, all comparisons, p < 0.0001, total number cell lines 747. ns = not significant, *p < 0.05, **p < 0.01, ***p < 0.001, ****p < 0.0001. [file 13046_2021_2124_MOESM4_ESM.pdf]

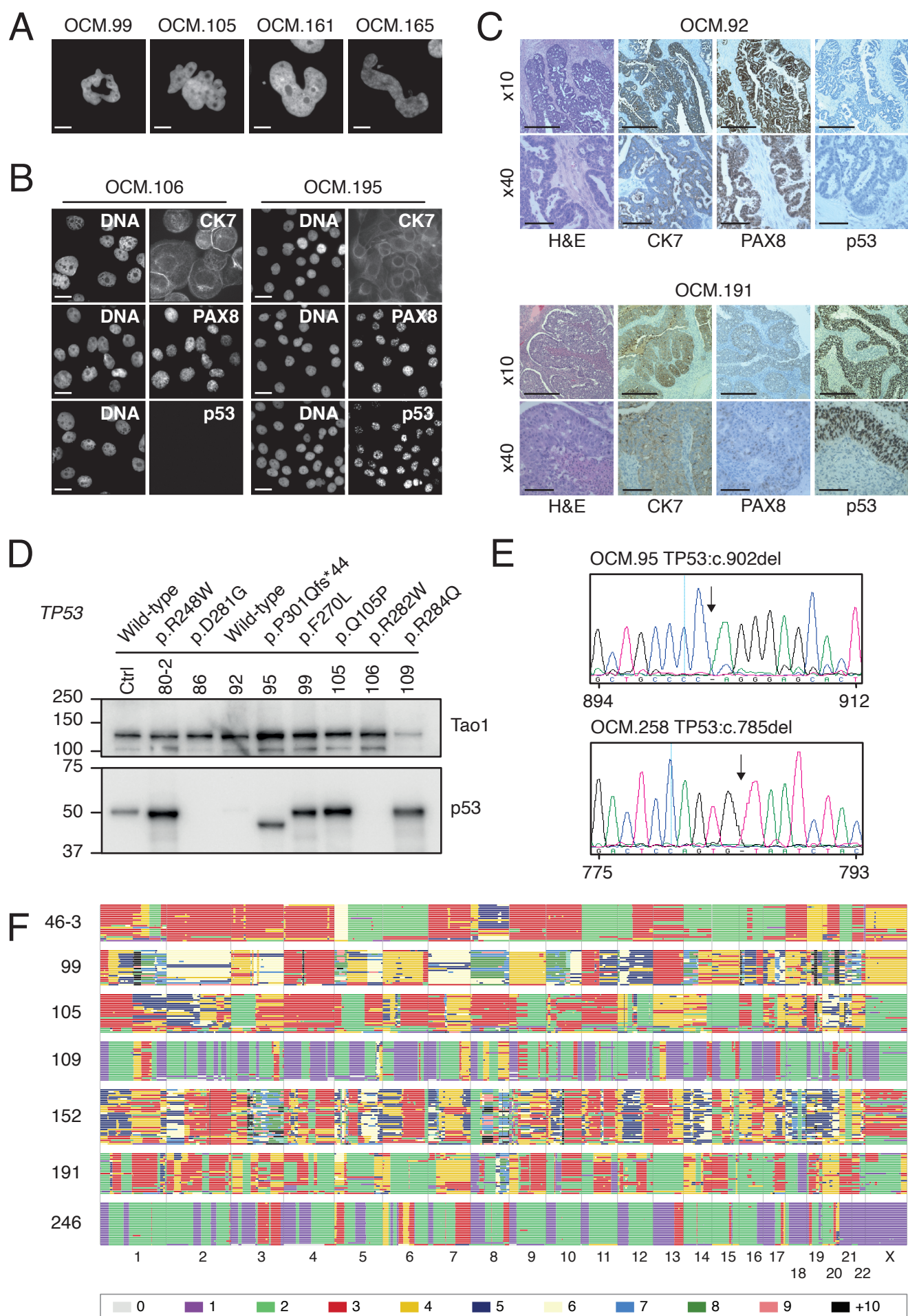

Figure S5

Supplement: Supplementary file 5 — Additional file 5: Fig. S5. Validation of patient-derived OCMs as bona fide models of HGSOC. (A) Representative images of severely atypical nuclei seen across OCMs. Scale bar: 10 μm. (B) Representative images of CK7 and PAX8, and p53 mutation-type (OCM.106: absent nuclear expression; OCM.195: strong/diffuse nuclear expression, involving > 80% tumour cell nuclei) by immunofluorescence staining. Scale bar: 20 μm. (C) Representative images of CK7, PAX and p53 mutation-type (OCM.92: absent nuclear expression; OCM.191: strong/diffuse nuclear expression, involving > 80% tumour cell nuclei) by immunohistochemistry staining from archival tumour blocks. Scale bar: 500 μm (× 10 magnification) and 100 μm (× 40 magnification). (D) Representative p53 immunoblot e.g. showing absent (OCM.86) and strong p53 bands (OCM.105). The control well represents stromal cells from the patient sample associated with OCM.237. Tao1 serves as loading control. (E) Representative somatic TP53 variants detected in OCMs. (F) Exemplar images of genome-wide chromosome copy-number profiles determined by single-cell whole-genome sequencing showing aneuploidies and rearranged chromosomes in tumour cells. Each row represents a single cell, with chromosomes plotted as columns and colours depicting copy-number state. See also Supplementary Table 1. [file 13046_2021_2124_MOESM5_ESM.pdf]

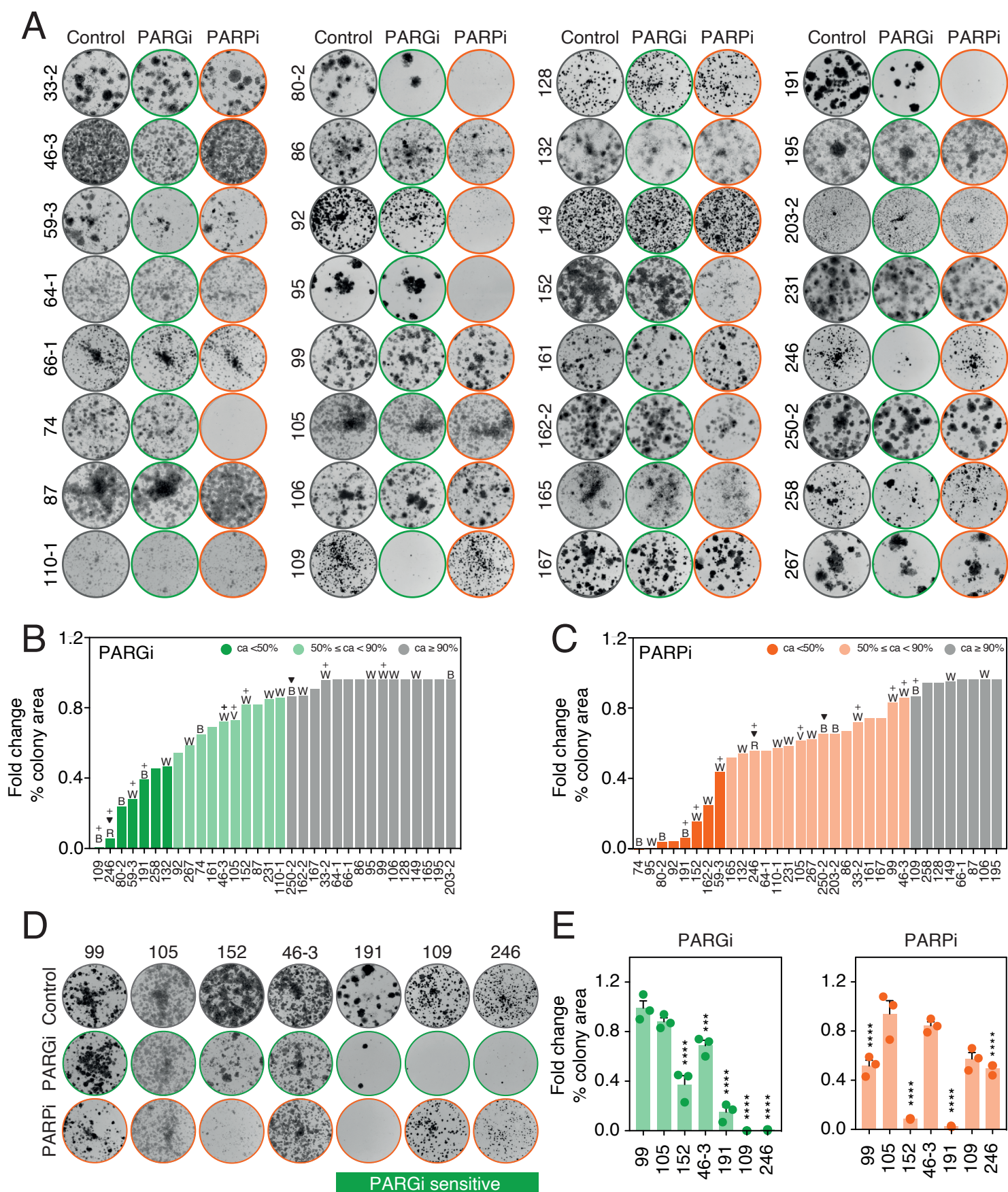

Figure S6

Supplement: Supplementary file 6 — Additional file 6: Fig. S6. Living biobank screen demonstrated broad range of PARGi and PARPi sensitivity. (A) Colony formation following 96 h of treatment with 1 μM PARGi or 1 μM PARPi or DMSO (Control). (B) Quantification of ca from (A) following 1 μM PARGi (B) or 1 μM PARPi (C), normalised to DMSO-treated cells (Control) and represented as fold-change. BRCA status (germline, or, where available, OCM) indicated as follows: + = BRCA status of OCM; W=BRCA1/2 wild-type; B=BRCA1/2 mutation; V=BRCA1/2 variant of uncertain clinical significance; R = putative BRCA1/2 reversion; ▼=prior PARPi therapy. Single technical replicate. (D) Exemplar images of colony formation following continuous treatment with 1 μM PARGi or 1 μM PARPi or DMSO (Control). Representative images of 3 biological replicates. (E) Quantification of ca from (D) normalised to DMSO-treated cells (Control) and represented as fold-change. Mean of 3 biological replicates. Error bars represent SEM. See also Supplementary Table 2 and Fig. 5A, B. ***p < 0.001, ****p < 0.0001. [file 13046_2021_2124_MOESM6_ESM.pdf]

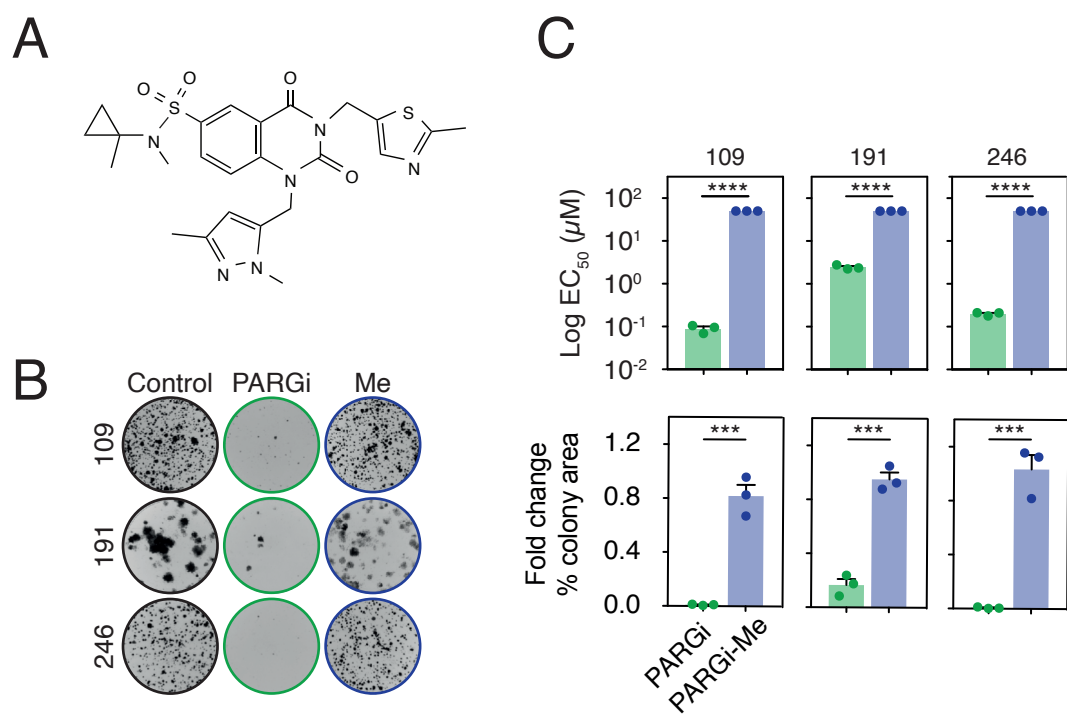

Figure S7

Supplement: Supplementary file 7 — Additional file 7: Fig. S7. On-target inhibition of PARG in PARGi-sensitive OCMs. (A) Chemical structure of inactive small molecule analog of PARGi, PARGi-Me (PDD00031704). (B) Colony formation of PARGi-sensitive OCMs (109, 191 and 246) following continuous treatment with 1 μM PARGi or 1 μM PARGi-Me or DMSO (Control). Representative images of 3 biological replicates. (C) Upper panel – Proliferative Log EC50 values for PARGi and PARGi-ME for PARGi-sensitive OCMs. Mean of 3 biological replicates. Error bars represent SEM. Statistics: Unpaired t-test of PARGi versus PARGi-Me. Lower panel – Quantification of ca from (B) normalised to DMSO-treated cells (Control) and represented as fold-change. Mean of 3 biological replicates. Statistics: Unpaired t-test of PARGi versus PARGi-Me. Error bars represent SEM. ***p < 0.001, ****p < 0.0001. [file 13046_2021_2124_MOESM7_ESM.pdf]

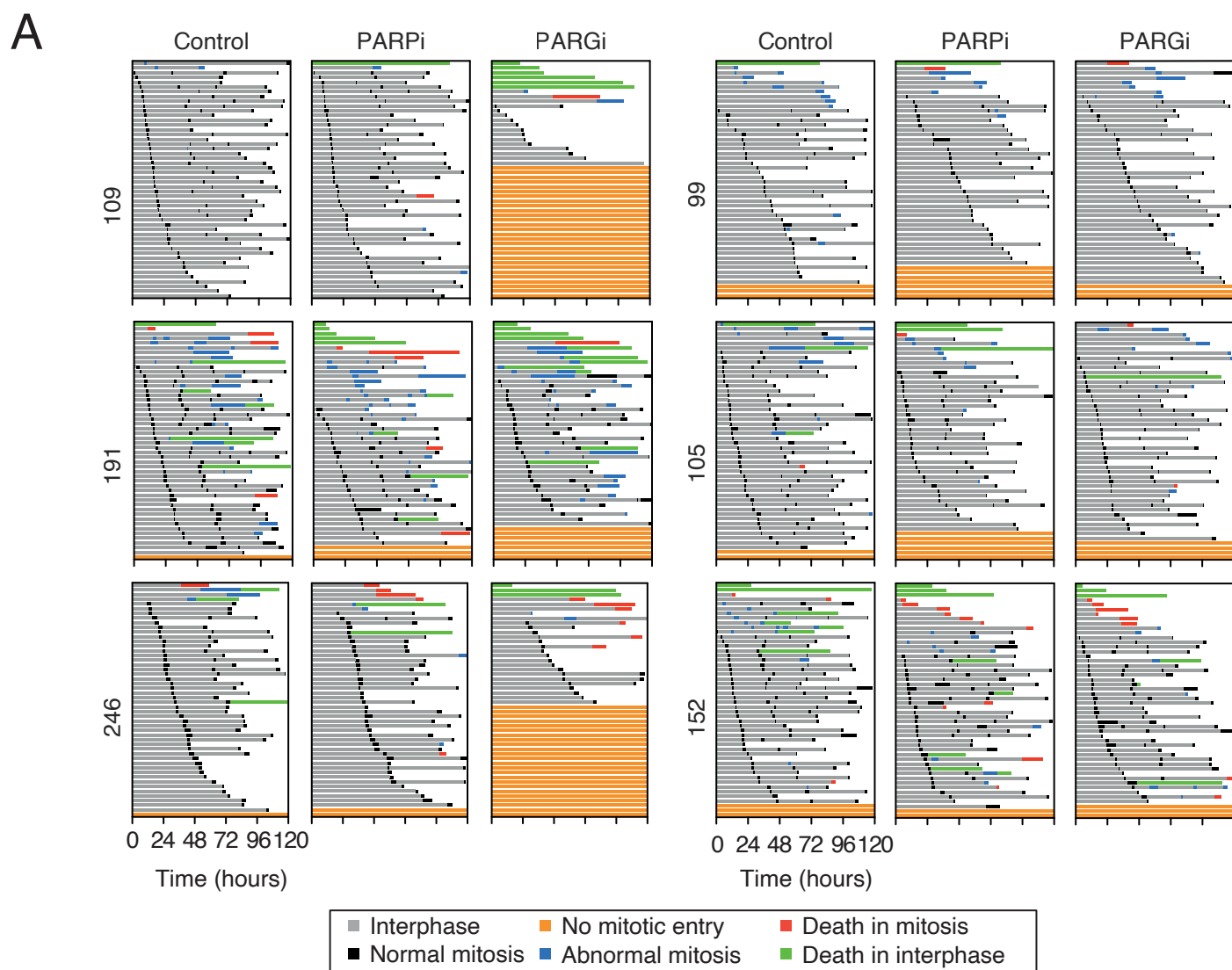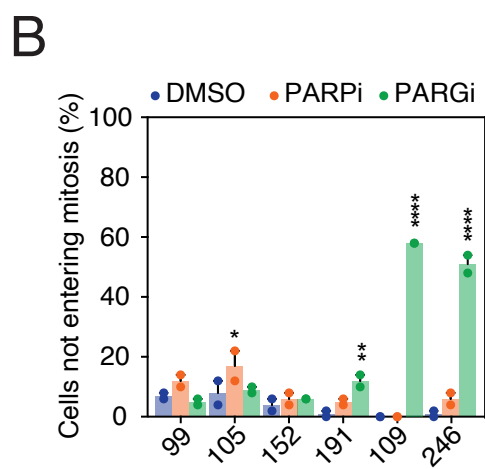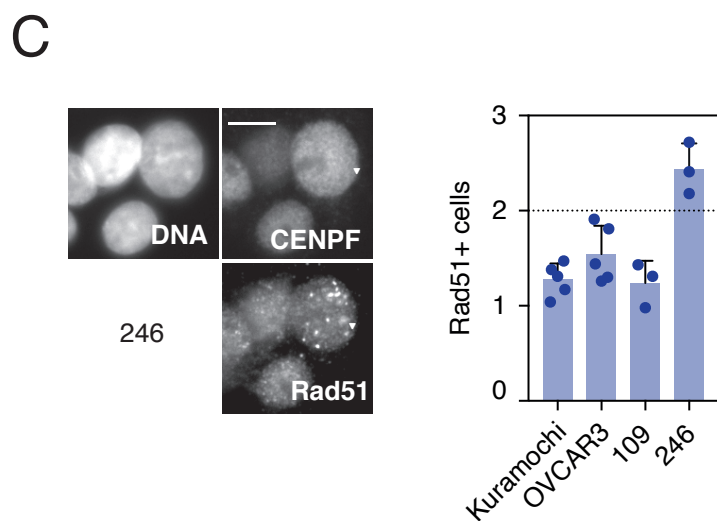

Figure S8

Supplement: Supplementary file 8 — Additional file 8: Fig. S8. PARGi suppresses mitotic entry in sensitive OCMs. (A) Cell fate profiling, showing cell behaviour over 120 h treatment with 1 μM PARGi or DMSO (Control). Each horizontal line represents a single cell, with the colours indicating cell behaviour. Following mitosis, one daughter cell was chosen at random to continue the analysis. No mitotic entry does not include death in interphase where mitosis does not take place. Abnormal mitosis includes cell division, abnormalities such as tripolar cell divisions, binuclear daughter cells and division of binuclear cells. Slippage is recorded where cells enter mitosis, then exit without division. Fusion was recorded where daughter cells appear to separate but subsequently join back together. Representative of 2 biological replicates. (B) Number of cells that fail to enter mitosis over 120 h (maximum n = 50) following 1 μM PARGi, 1 μM PARPi or DMSO (Control). Statistics: 2-way ANOVA with Dunnett’s multiple comparisons test, selected comparisons were between PARGi or PARPi treated cells versus DMSO control. Mean of 2 biological replicates. Error bars represent SEM. (C) Exemplar image of CENPF (indicating G2 cells) and Rad51 immunofluorescence staining of OCM.246 after 2 Gy X-ray ionising radiation followed by 24 h PARPi, ▼ indicates an HRP cell (positive for CENPF with > 5 Rad51 foci). Scale bar: 10 μm. Bar chart shows fold-change in % [Rad51 + CENPF+ cells/CENPF+ cells] in DMSO-treated cells versus cells treated with 2 Gy X-ray ionising radiation followed by 24 h 1 μM PARPi. Dotted line indicates a 2-fold change, above which cells are considered HRP (below it HRD). [file 13046_2021_2124_MOESM8_ESM.pdf]

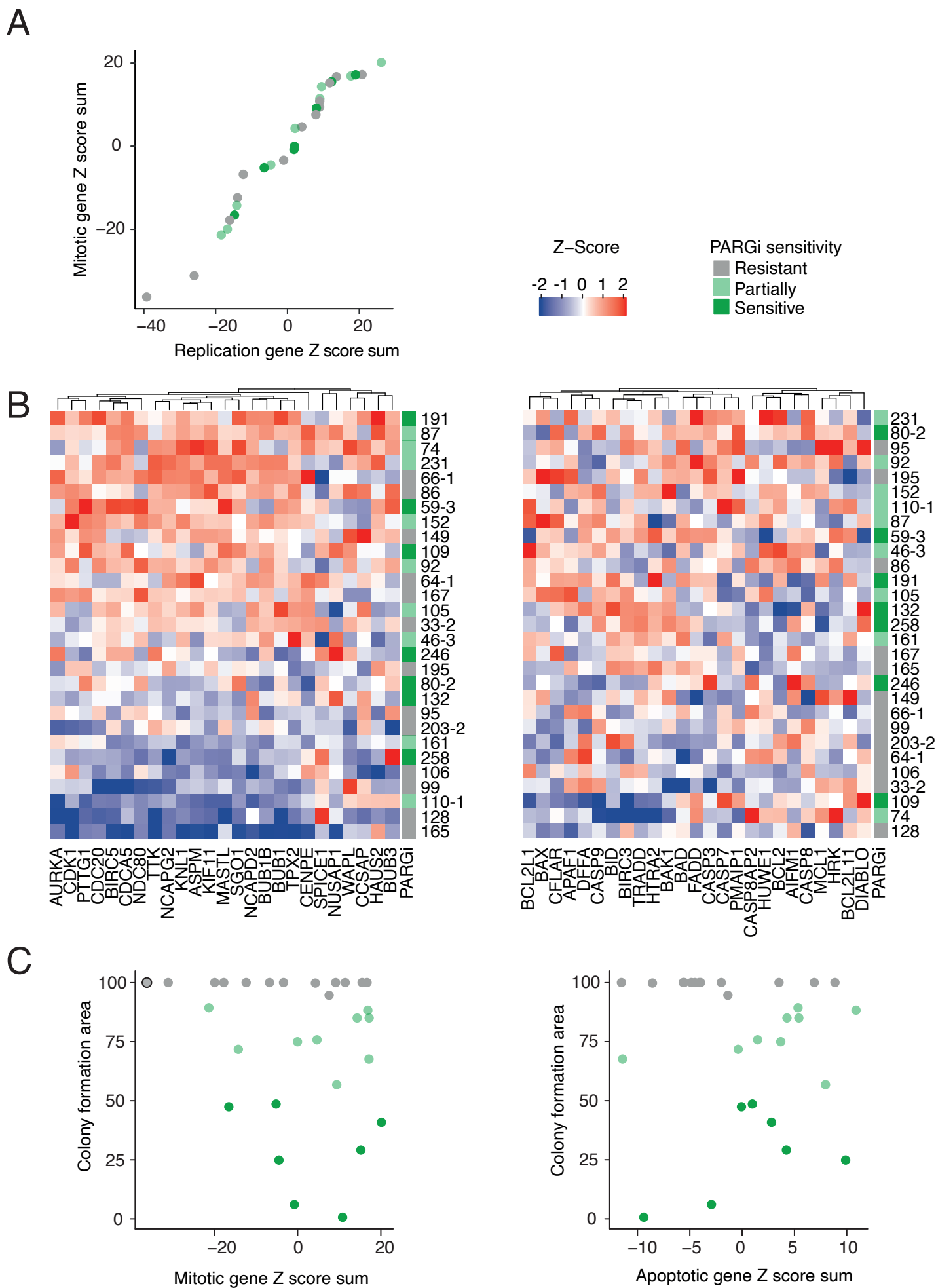

Figure S9

Supplement: Supplementary file 9 — Additional file 9 Fig. S9. Low expression of mitotic or apoptotic gene sets does not identify OCMs sensitive to PARGi. (A) XY plot showing correlation between sum of Z scores for mitotic and DNA replication genes for each OCM (p < 0.0001, Pearson’s R2 = 0.774). (B) Heatmaps showing RNAseq of 29 OCMs ranked by sum of z-scores for 25 mitotic (left) and apoptotic (right) genes where red = high expression; white = average expression; and blue = low expression. (C) XY plots showing colony formation area and the sum of z-scores for the expression of 25 mitotic (left) and apoptotic (right) genes from RNAseq of 29 OCMs. PARGi sensitivity as determined by colony formation assay (see Fig. S6B). Note: RNAseq was unavailable for OCMs 267, 250–2, and 162–2. [file 13046_2021_2124_MOESM9_ESM.pdf]
