# Supplementary material for: Replication catastrophe is responsible for intrinsic PAR glycohydrolase inhibitor-sensitivity in patient-derived ovarian cancer models
Source: J Exp Clin Cancer Res. 2021 Oct 16;40:323. doi: 10.1186/s13046-021-02124-0 (PMC8520217; doi:10.1186/s13046-021-02124-0)
Supplement: Supplementary file 10 — Additional file 10: Supplementary Table 1. Clinical data, OCMs and primary tumour blocks. The table outlines the clinical data for the 25 new HGSOC OCMs screened for PARPi and PARGi sensitivity. Key: dx, diagnosis; FIGO, International Federation of Gynecology and Obstetrics; gBRCAm, germline BRCA1/2 mutation; VUS, variant of uncertain clinical significance; CTx, chemotherapy; Ref, platinum-refractory disease (tumour progression during or within 4 weeks of completing platinum therapy); Res, platinum-resistant (tumour progression between 4 weeks and 6 months from completing platinum therapy); Sens, platinum-sensitive (tumour progression ≥6 months from completing platinum therapy). CN, chemonaïve; OS, overall survival; mo, months; WT, wild-type; FR, frameshift; NS, nonsense; IF, immunofluorescence; IHC, immunohistochemistry; For IF and IHC: CK7 and PAX8, coloured box (present), white box (absent), S, strong; P, patchy; F, focal; W, weak. p53 is either mutant-type (strong/diffuse nuclear staining; darker coloured box), wild-type (lighter colour box) or absent nuclear staining (white box). NE, not evaluable (antibody failed); block unavailable (grey box). IB, immunoblotting; For IB: +(sm), band present but at lower than 53 kDa; +(S), strong band present; *At the time of the research biopsy; †Histologically re-classified from HGSOC to intermediate grade (grade 2/moderately differentiated) serous adenocarcinoma following tumour block analysis; ‡cell agar block only, histologically re-classified from HGSOC to suspicion of adenocarcinoma arising from the gynaecological tract. Clinical data for previously characterised OCMs are published [13], and not repeated here. Supplementary Table 2. BRCA1/2 variants detected in OCMs. The table outlines the BRCA1/2 variants detected in the panel of 7 OCMs screened for PARGi and PARPi sensitivity using colony survival and cell proliferation assays. Variants are described using Human Genome Variation Society (HGVS) nomenclature. Key: FR, f [file 13046_2021_2124_MOESM10_ESM.pdf]

Supplementary Tables

Supplementary Table 1. Clinical data, OCMs and primary tumour blocks

OCM	Patient demographics						*Ex vivo* culture					Primary tumour		
							IF			IB	Sanger	IHC		
	FIGO stage	Histology	g*BRCA*m	Prior lines of CT_x*	Platinum Sensitivity *	OS (mo)	CK7	PAX8	p53	p53	*TP53m* RT-PCR	CK7	PAX8	p53
80-2	3C	HGSOC	*BRCA1*	2	Sens	-	P			+(S)	p.R248W			
86	4B	HGSOC	Unknown	3	Res	25.0		W		-	p.D281G			
92	3C	HGSOC	Unknown	1	Res	9.6		W		-	WT	P		
95	3C	HGSOC	WT	4	Res	39.7				+(sm)	FR			
99	3C	HGSOC	*BRCA2* VUS	0	CN	39.5	P			+	p.F270L			
105	3C	HGSOC	*BRCA1* VUS	3	Ref	21.4				+(S)	p.Q105P	F		
106	3C	HGSOC	WT	7	Res	62.1				-	p.R282W			
109	4B	HGSOC	Unknown	3	Res	26.8				+(S)	p.R248Q	F		
110-1	3C	HGSOC	WT	0	CN	7.7				-	p.R248Q			
128	3C	HGSOC	Unknown	2	Sens	25.9				-	FR			
132	3C	HGSOC	WT	2	Res	-				-	p.R248W			NE
149	3C	HGSOC	WT	5	Res	57.6		W		+	p.R248L			
152	3C	HGSOC†	Unknown	5	Res	55.9				+	p.Y220C			
161	4A	HGSOC	Unknown	2	Res	29.4		W		-	WT			
162-2	3C	HGSOC	WT	4	Res	39.9		W		-	Exon 6 deletion	S		
165	3C	HGSOC	Unknown	3	Res	17.7				+	p.Y220C	S		
167	3C	HGSOC	Unknown	3	Res	29.8		W		+	WT			
191	3A	HGSOC	*BRCA1*	2	Res	17.8				+(S)	p.R248Q		P	
195	4A	HGSOC‡	Unknown	0	CN	10.2		W		+(S)	WT	‡	‡	‡
203-2	3C	HGSOC	*BRCA1*	6	Res	69.5				+	p.R342P			
231	3C	HGSOC	WT	0	CN	-				+	p.R248G			
246	4A	HGSOC	*BRCA2*	4	Res	61.3				-	NS			
250-2	3C	HGSOC	*BRCA1*	3	Res	-				+(S)	p.I195N			
258	3C	HGSOC	Unknown	1	Res	14.2				+(sm)	FR			
267	4B	HGSOC	WT	2	Res	25.4				+(S)	p.Y220C			

Supplementary Table 2. *BRCA1/2* variants detected in OCMs

OCM	*Germline BRCA1/2* variant	OCM			
		***BRCA1/2* variant**	**Variant type**	**Classification**	**VAF**
46-3	Unknown	WT	-	-	-
99	*BRCA2:*c.316+13A>G (p.?) Intronic variant (VUS)	WT	-	-	-
105	*BRCA1*:c.5452G>A p.(Asp1818Asn) Missense (VUS)	*BRCA1:*c.5452G>A (p.Asp1818Asn)	MS	VUS	0.51
109	Unknown	*BRCA1*:c.329dup (p.Glu111GlyfsTer3)	FR	Pathogenic	0.92
152	Unknown	WT	-	-	-
191	*BRCA1*:c.3268C>T (p.Gln1090Ter) Nonsense	*BRCA1*:c.3268C>T (p.Gln1090Ter)	NS	Pathogenic	0.99
		*BRCA2:*c.4725C>G (p.Asp1575Glu)	MS	VUS	1.0
246	*BRCA2:*c.5946del (p.Ser1982ArgfsTer22) Frameshift	*BRCA2:*c.5087_6841del (p.Ile1697_Gly2281del)	In-frame deletion	Putative reversion	Sanger
		*BRCA2:*c.4946_6841del (p.Lys1649_Gly2281delinsArg)	In-frame deletion	Putative reversion	Sanger

Supplementary Table 3. EC_{50} values for OCMs for the inhibitors/compounds tested.

OCM	EC_{50} values				
	PARGi (μM)	Olaparib (μM)	Niraparib (μM)	Cisplatin (μM)	Paclitaxel (nM)
46-3	NC	7.7±0.9	-	0.9±0.1	5.6±0.3
99	NC	6.2±1.3	-	0.8±0.2	7.6±1.2
105	NC	16.1±9.0	-	1.3±0.1	4.4±0.5
109	0.09±0.01	14.5±1.4	10.3±3.1	1.1±0.1	6.9±1.1
152	NC	2.9±1.3	-	1.2±0.2	3.3±0.2
191	3.2±0.1	2.5±0.2	-	0.7±0.3	5.8±0.9
246	0.2±0.1	3.9±0.4	4.4±0.5	0.7±0.3	27.1±4.9
